# Major Bleeding Risk in Atrial Fibrillation Patients Co-Medicated With Non-Vitamin K Oral Anticoagulants and Antipsychotics

**DOI:** 10.3389/fphar.2022.819878

**Published:** 2022-04-14

**Authors:** Chiung-Mei Chen, Kuo-Hsuan Chang, Chun-Li Wang, Hui-Tzu Tu, Yu-Tung Huang, Hsiu-Chuan Wu, Chien-Hung Chang, Shang-Hung Chang

**Affiliations:** ^1^ Department of Neurology, Chang Gung Memorial Hospital, Linkou Medical Center, Taoyuan City, Taiwan; ^2^ College of Medicine, Chang Gung University, Taoyuan City, Taiwan; ^3^ Division of Cardiology, Department of Internal Medicine, Chang Gung Memorial Hospital, Linkou Medical Center, Taoyuan City, Taiwan; ^4^ Center for Big Data Analytics and Statistics, Chang Gung Memorial Hospital, Linkou Medical Center, Taoyuan City, Taiwan; ^5^ Graduate Institute of Nursing, Chang Gung University of Science and Technology, Taoyuan City, Taiwan

**Keywords:** major bleeding risk, non-vitamin K oral anticoagulant, antipsychotics, non-valvular atrial fibrillation, combined medication

## Abstract

Major bleeding risks associated with non-vitamin K oral anticoagulants (NOACs) used with and without concurrent antipsychotics in patients with non-valvular atrial fibrillation (AF) were assessed. A total of 98,863 patients with non-valvular AF receiving at least one NOAC prescription from Taiwan’s National Health Insurance database were enrolled. Major bleeding was defined as a primary diagnosis of intracranial or gastrointestinal hemorrhage or bleeding at other sites. The adjusted incidence rate difference (AIRD) per 1,000 person-years and adjusted rate ratio of major bleeding were estimated using Poisson regression and inverse probability of treatment weighting using the propensity score. A total of 8,037 major bleeding events occurred during 705,521 person-quarters with NOAC prescriptions. Antipsychotics were used in 26.35% of NOAC-exposed patients. Compared to using NOAC alone, co-medication of either typical (AIRD: 79.18, 95% confidence interval [CI]: 70.63–87.72) or atypical (AIRD: 40.5, 95% CI: 33.64–47.35) antipsychotic with NOAC had a significant increase in the adjusted incidence rate per 1,000 person-years of major bleeding. The concomitant use of a NOAC with chlorpromazine (AIRD: 103.87, 95% CI: 51.22–156.52), haloperidol (AIRD: 149.52, 95% CI: 125.03–174.00), prochlorperazine (AIRD: 90.43, 95% CI: 78.55–102.32), quetiapine (AIRD: 44.6, 95% CI: 37.11–52.09), or risperidone (AIRD: 41.55, 95% CI: 22.86–60.24) (All *p <* 0.01) showed a higher adjusted incidence rate of major bleeding than using NOACs alone. The concomitant use of typical (chlorpromazine, haloperidol, or prochlorperazine) or atypical (quetiapine or risperidone) antipsychotic with NOACs was associated with a significantly increased risk of major bleeding.

## Introduction

Atrial fibrillation (AF) is a major cause of thromboembolic stroke, morbidity, mortality, and healthcare expenditure among elder populations (Zimetbaum, 2017). Non-vitamin K antagonist oral anticoagulants (NOACs), an alternative to vitamin K antagonists (VKAs) to prevent thromboembolic stroke in patients with non-valvular AF, have been recommended as the preferred choice compared to VKAs due to NOACs’ associations with lower rates of major bleeding, easier use, and fewer drug–drug interactions (DDIs) than VKAs (Ruff et al., 2014). An observation study also showed that the use of direct oral anticoagulants to treat AF patients increased during the study years, accompanied by substantially declined 1-year hospitalization for ischemic stroke and hemorrhagic strokes. Particularly, the total medical cost per AF patient in the last study year was lower than that in the previous years (Maggioni et al., 2020). However, most of the non-valvular AF patients treated with NOACs are polypharmacy patients, and DDIs remain an important factor associated with the increased bleeding risk (Ajam et al., 2020). NOACs are substrates of drug efflux pump P-glycoprotein (P-gp) and are metabolized mainly by cytochrome P450 (CYP)3A4. The drugs sharing these metabolic pathways, which include anti-epileptics, anti-arrhythmic medications, and antibiotics, are associated with the increased bleeding risk *via* DDIs with NOACs (Chang et al., 2017; Stöllberger, 2017; Ajam et al., 2020; Wang et al., 2020). Several studies have shown AF increases risks of cognitive decline and dementia (Diener et al., 2019). Psychotic symptoms among elderly patients with schizophrenia spectrum, delirium, affective disorders, dementia, and stroke are common (Tampi et al., 2019). Antipsychotics are frequently used to treat psychotic disorders among late-life patients (Gareri et al., 2014; Kirkham et al., 2017). Antipsychotics are classified into typical (first-generation) and atypical (second-generation) groups (Meltzer, 2013). The frequency of the combined use of NOACs and antipsychotics in non-valvular AF patients, which are mostly elderly, is unknown. Many antipsychotics are CYP3A4 and/or P-gp inhibitors (Boulton et al., 2002; El Ela et al., 2004; Ayano, 2016), but the bleeding risk in NOAC users co-medicated with antipsychotics has yet to be examined.

To determine the bleeding risk of co-administration of antipsychotics with NOACs, we used a nationwide cohort of patients with non-valvular AF to estimate the bleeding risk associated with the concomitant therapy of antipsychotics in NOAC users.

## Materials and Methods

### Ethical Approval

The institutional review board at Chang Gung Memorial Hospital provided ethical approval, and valid data use agreements were in place.

### Population of Patients

**FIGURE 1:**
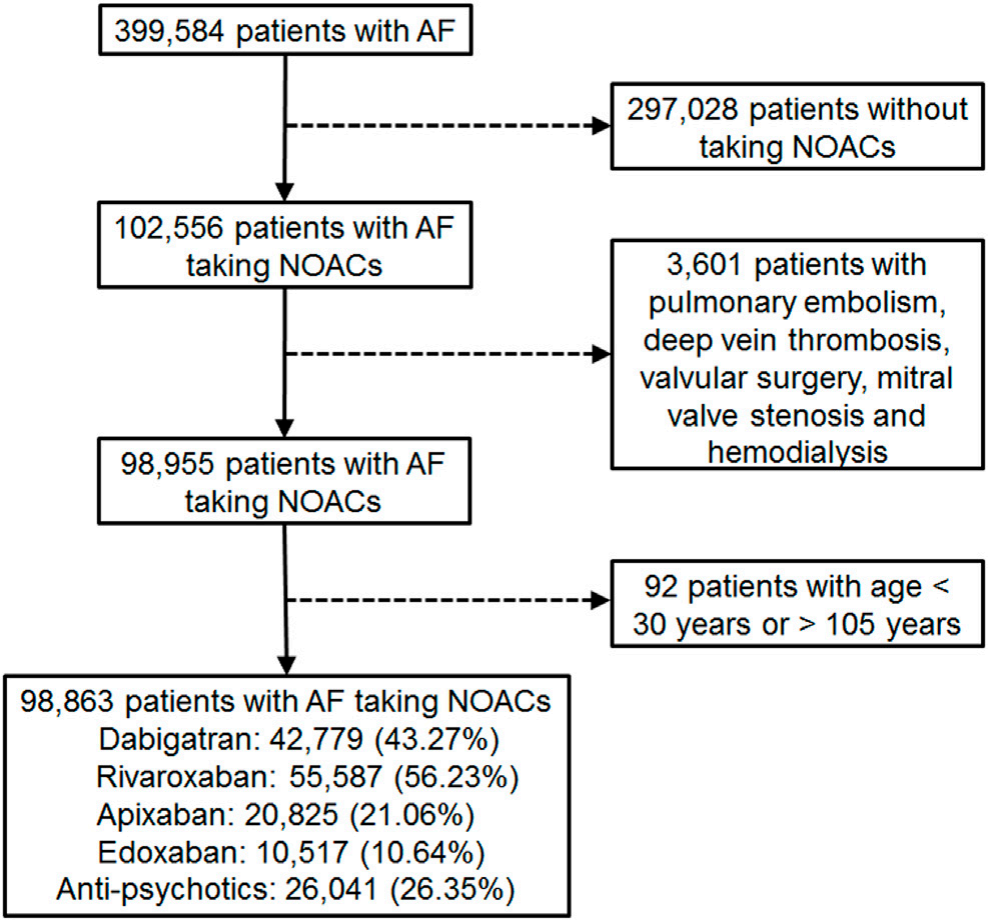
Enrollment of patients with nonvalvular atrial fibrillation (AF) with medication of non-vitamin K antagonist oral anticoagulants (NOACs).

Patient data in this retrospective cohort study were retrieved from the National Health Insurance Research Database (NHIRD), including outpatient, inpatient, and prescription records accessed through the Health and Welfare Data Science Center, Ministry of Health and Welfare, Taiwan. The NHIRD is claims-based reimbursement data collected from the NHI administration. The NHI system was established in 1995 and covers more than 99.6% of Taiwan’s residents (over 24 million population), including every citizen with official residency or every person with a foreign nationality living in Taiwan with an Alien Resident Certificate. All patients’ identities were encrypted. Diagnoses/procedures were identified by the International Classification of Diseases, 9th Revision, Clinical Modification (ICD-9-CM) codes from 1997 through 2015 and the International Classification of Diseases, 10th Revision, Clinical Modification (ICD-10-CM) codes, from 2016. All patients (outpatients and/or inpatients) having two or more consecutive records of AF diagnosis (ICD-9-CM code 427.31 or ICD-10-CM code I48), which is to avoid misclassification (Chang et al., 2017), and NOAC prescriptions for more than 28 days from 1 June 2012 to 31 December 2017 were recruited (Figure 1). The NOACs included dabigatran (identified by the Anatomical Therapeutic Chemical [ATC] classification system code, B01AE07), rivaroxaban (ATC code, B01AF01), apixaban (ATC code, B01AF02), and edoxaban (ATC code, B01AF03). The index date was defined as the first NOAC prescription. Patients were excluded if they were diagnosed with pulmonary embolism, deep vein thrombosis, mitral stenosis, joint replacement, or valvular surgery, within 6 months before the index date, or diagnosed with end-stage renal disease, or <30 or >105 years of age. Patients were followed until death, withdrawal from the NHI, or the end of the study (31 December 2017), whichever occurred first.

### Follow-Up of Patients

We divided each calendar year into four quarters for each patient. The analytic unit was one person-quarter. Person-quarters were used because medications for chronic illnesses were refilled with a maximum length of 3 months under the NHI reimbursement policy. Medications and covariates were assessed for each person-quarter, which simplified the assessment of the complex prescription pattern of NOACs and concurrent drugs. Person-quarters exposed to NOACs with or without concurrent antipsychotics were identified (Supplementary Figure S1). ATC classification system codes were used to identify drug prescriptions. The major bleeding risks of person-quarters exposed to NOACs and concurrent antipsychotics were compared with person-quarters exposed to NOACs alone. Only antipsychotics prescribed for more than 0.18% of assessed patients were included in the study. These antipsychotics were further classified into typical (chlorpromazine, droperidol, haloperidol, flupentixol, and prochlorperazine) and atypical (aripiprazole, olanzapine, quetiapine, and risperidone) groups. Flupentixol/melitracen combined medication was also analyzed as being in the typical group. Person-quarters with concomitant prescriptions of NOACs and warfarin or two NOACs were excluded.

### Major Outcomes and Covariates

The primary outcome was major bleeding, identified by ICD codes upon a visit to the emergency department of the hospital, with a primary diagnosis of intracranial, gastrointestinal, or bleeding at other sites (Supplementary Table S1). Each person-quarter included only one major bleeding event, and traumatic hemorrhage was excluded. This is to avoid overestimating the bleeding risk when there are two major bleeding events within one person-quarter. Covariates, including demographics, comorbidities, medications, and the expense of medical utilization, were assessed for each person-quarter relevant to the first date of enrollment of patients (Tables 1, 2 and Supplementary Tables S2, S3). Demographics included age, gender, income level, residency, occupation, and the number of outpatient visits. Factors of the Charlson Comorbidity Index (Charlson et al., 1987), CHA2DS2-VASc score (Lip et al., 2010), HAS-BLED (hypertension, abnormal kidney or liver function, stroke, bleeding history, and antiplatelet drug or alcohol use) score (Pisters et al., 2010), other diseases, and medications prescribed longer than 28 days in each person-quarter potentially related to hemorrhage or interacting with NOACs were also included as covariates (Tables 1, 2 and Supplementary Table S3). The HAS-BLED score was calculated, as previously described (Tsai et al., 2020). CHA2DS2-VASc and HAS-BLED scores were estimated by using diagnoses collected in the database. The NHI of Taiwan covers almost all services that can be provided by a health system: from dental care to parturition, from Western medicine to traditional Chinese medicine, and from preventive services to elderly home care. Therefore, only a few self-pay medical data were not included in the NHIRD; the data for our study subjects should be comprehensive. Abnormal kidney or liver function was defined by ICD-9-CM codes rather than by laboratory values. Because the international normalized ratio (INR) for warfarin was not available in the NHIRD, the INR was excluded from scoring in the present study.

**TABLE 1:** Demographic analysis of patients with the use of NOACs.

	NOAC users (*n* = 98,863)	Without antipsychotics (*n*=72,817)	With antipsychotics (*n* =26,046)
Age (years)	74.69 ± 10.52	73.41 ± 10.74	76.68 ± 9.84
Male (%)	55,610 (56.25%)	42,599 (58.50%)	13,011 (49.95%)
Residence			
Urban (%)	52,055 (52.65%)	38,932 (53.46%)	13,123 (50.39%)
Suburban (%)	32,384 (32.76%)	23,609 (32.42%)	8,775 (33.69%)
Rural (%)	13,761 (13.92%)	9,822 (13.39%)	3,939 (15.12%)
Unknown (%)	663 (0.67%)	454 (0.62%)	209 (0.80%)
Occupation			
Dependents of the insured individuals (%)	38,834 (39.28%)	28,630 (39.32%)	10,204 (39.18%)
Civil servants, teachers, military personnel, and veterans (%)	11,812 (11.95%)	8,193 (11.25%)	3,619 (13.90%)
Nonmanual workers and professionals (%)	5,706 (5.77%)	4,867 (6.68%)	839 (3.22%)
Manual workers (%)	31,636 (32.00%)	22,831 (31.35%)	8,805 (33.81%)
Others (%)	10,719 (10.84%)	8,416 (11.56%)	2,303 (8.84%)
Income			
Quantile 1 (%)	28,810 (29.14%)	20,730 (28.47%)	8,080 (31.02%)
Quantile 2 (%)	1,475 (1.49%)	1,063 (1.46%)	412 (1.58%)
Quantile 3 (%)	41,130 (41.6%)	30,004 (41.20%)	11,126 (42.72%)
Quantile 4 (%)	9,738 (9.85%)	7,376 (10.13%)	2,362 (9.07%)
Quantile 5 (%)	17,492 (17.69%)	13,496 (18.53%)	3,996 (15.34%)
Unknown (%)	218 (0.22%)	148 (0.20%)	70 (0.27%)
CHAD_2_DS_2_-VASc score	4.34 ± 1.75	4.08 ± 1.75	4.75 ± 1.68
HAS-BLED score	3.04 ± 1.19	2.91 ± 1.19	3.24 ± 1.16
Anemia (%)	15,520 (15.7%)	10,054 (13.81%)	5,466 (20.99%)
Acute pancreatitis (%)	1,639 (1.66%)	1,091 (1.50%)	548 (2.10%)
Acute appendicitis (%)	1,601 (1.62%)	1,155 (1.59%)	446 (1.71%)
Cancer (%)	14,064 (14.23%)	9,735 (13.37%)	4,329 (16.62%)
Metastatic solid tumor (%)	1,334 (1.35%)	894 (1.23%)	440 (1.69%)
Cardiovascular diseases			
Hypertension (%)	86,688 (87.68%)	62,772 (86.20%)	23,916 (91.82%)
Myocardial infarction (%)	6,507 (6.58%)	4,622 (6.35%)	1,885 (7.24%)
Congestive heart failure (%)	51,300 (51.89%)	36,435 (50.04%)	14,865 (57.07%)
Peripheral vascular disease (%)	13,896 (14.06%)	9,428 (12.95%)	4,468 (17.15%)
Peripheral arterial occlusive disease (%)	2,677 (2.71%)	1,844 (2.53%)	833 (3.20%)
Percutaneous coronary intervention (%)	8,660 (8.76%)	6,185 (8.49%)	2,475 (9.50%)
Coronary artery bypass surgery (%)	1,121 (1.13%)	851 (1.17%)	270 (1.04%)
Chronic kidney disease (%)	26,568 (26.87%)	18,427 (25.31%)	8,141 (31.26%)
Gastrointestinal and hepatic diseases			
Peptic ulcer disease (%)	56,455 (57.10%)	39,028 (53.60%)	17,427 (66.91%)
Mild liver disease (%)	36,580 (37.00%)	26,524 (36.43%)	10,056 (38.61%)
Moderate or severe liver disease (%)	334 (0.34%)	221 (0.30%)	113 (0.43%)
Human immunodeficiency virus infection (%)	25 (0.03%)	15 (0.02%)	10 (0.04%)
Intestinal obstruction without mention of hernia (%)	5,147 (5.21%)	3,224 (4.43%)	1,923 (7.38% )
Metabolic disease			
Diabetes mellitus (%)	41,790 (42.27%)	29,752 (40.86%)	12,038 (46.22%)
Diabetes with complications (%)	14,745 (14.91%)	10,201 (14.01%)	4,544 (17.45%)
Neurological diseases			
Cerebral vascular disease (%)	50,422 (51.00%)	36,011(49.45%)	14,411(55.33%)
Ischemic stroke (%)	37,610 (38.04%)	25,706 (35.30%)	11,904 (45.70%)
Transient ischemic attack (%)	12,915 (13.06%)	8,630 (11.85%)	4,285 (16.45 %)
Hemiplegia and paraplegia (%)	5,096 (5.15%)	3,268 (4.49%)	1,828 (7.02%)
Dementia (%)	11,427 (11.56%)	6,280 (8.62%)	5,147 (19.76%)
Epilepsy (%)	2,793 (2.83%)	1,886 (2.59%)	907 (3.48 %)
Pulmonary disease			
Chronic pulmonary disease (%)	52,619 (53.22%)	37,923 (52.08%)	14,696 (56.42%)
Chronic obstructive pulmonary disease (%)	47,585 (48.13%)	32,697 (44.90%)	14,888 (57.16%)

NOACs, non-vitamin K oral anticoagulants.

**TABLE 2:** Medications during follow-up.

Medication	NOAC users (*n* = 98863)
Antibiotics and antifungal drugs	2,800 (2.83%)
Anticoagulants	98,863 (100%)
Apixaban	20,825 (21.06%)
Dabigatran	42,779 (43.27%)
Edoxaban	10,517 (10.64%)
Rivaroxaban	55,587 (56.23%)
Anti-epileptics	4,928 (4.98%)
Anti-hypertensives	59,324 (60.1%)
Anti-platelets	24,186 (24.46%)
Antipsychotics	26,046 (26.35%)
Typical	
Prochlorperazine	12,161 (12.30%)
Haloperidol	4,618 (4.67% )
Flupentixol	3,269 (3.30%)
FlupentixolM	3,201 (3.23%)
Chlorpromazine	685 (0.69%)
Droperidol	190 (0.19%)
Atypical	
Quetiapine	11,770 (11.90%)
Risperidone	2,326 (2.35%)
Olanzapine	751 (0.76%)
Aripiprazole	404 (0.41%)
Bisphosphate (%)	602 (0.61%)
Cardiovascular medications	36,505 (36.92%)
Cyclosporine (%)	49 (0.05%)
Glucocorticoid (%)	7,987 (8.08%)
Insulin (%)	6,236 (6.31%)
Lipid-lowering drugs	19,172 (19.39%)
Non-steroid anti-inflammatory drugs (%)	19,180 (19.4%)

NOAC, non-vitamin K oral anticoagulant.

### Statistical Models and Analysis

Confounding by indication from non-random treatment allocation for concurrent medications was a crucial feature when comparing the bleeding risk among NOAC-treated patients, with and without concurrent antipsychotics. This bias was accounted for by the propensity score, which estimated the probability that a patient was prescribed the concurrent antipsychotics during a person-quarter. The specific propensity score for each antipsychotic was calculated using logistic regression by the aforementioned covariates relevant to the first date of the person-quarter. Standardized differences were estimated to evaluate the balance of individual covariates before and after propensity score weighting. A negligible difference was defined as an absolute standardized mean difference <0.1. To account for intraindividual correlation across person-quarters, we used logistic regression, with a generalized estimating equations model to calculate the adjusted incidence rate that considered the inverse probability of treatment weighting by propensity scores. All regression analyses were performed separately for each combination, and person-quarters using NOAC alone were used as the reference category. Acute appendicitis was considered as a negative control of the outcome, and the association of acute appendicitis between NOACs with and without the concurrent antipsychotic use was analyzed. We further examined the effect of bleeding events within the first person-quarter of co-medication of NOACs and antipsychotics by excluding such events in an additional sensitivity analysis. Patients with missing data (<0.1% of NOAC users) were excluded. The analyses were performed by SAS (SAS Institute), version 9.4.

## Results

### Patient Characteristics

We identified a total of 98,863 AF patients undergoing NOAC therapy from 1 June 2012 to 31 December 2017 (Figure 1). The characteristics of the AF patients on the first date of the NOAC prescription are listed in Table 1, and the medications used during follow-up are listed in Table 2 and Supplementary Table S3. A certain portion of the patients was prescribed with different NOACs in different person-quarters during the study period. The mean age was 74.69 ± 10.52 years, and 56.25% of the studied population were male patients. The baseline average CHA2DS2-VASc score was 4.34 ± 1.75, and the average HAS-BLED score was 3.04 ± 1.19. More than 80% of the recruited patients had been diagnosed with hypertension, while congestive heart failure and cerebrovascular disease were seen in more than 50% of recruited patients. A total of 26.35% of NOAC-treated AF patients had concurrent use of antipsychotics during the follow-up (Figure 1). The most common antipsychotics prescribed with concurrent NOACs were prochlorperazine (12.30%), followed by quetiapine (11.90%), haloperidol (4.67%), flupentixol (3.30%), flupentixol/M (3.23%), risperidone (2.35%), olanzapine (0.76%), chlorpromazine (0.69%), and aripiprazole (0.41%) (Table 2).

### Major Bleeding Events

**FIGURE 2:**
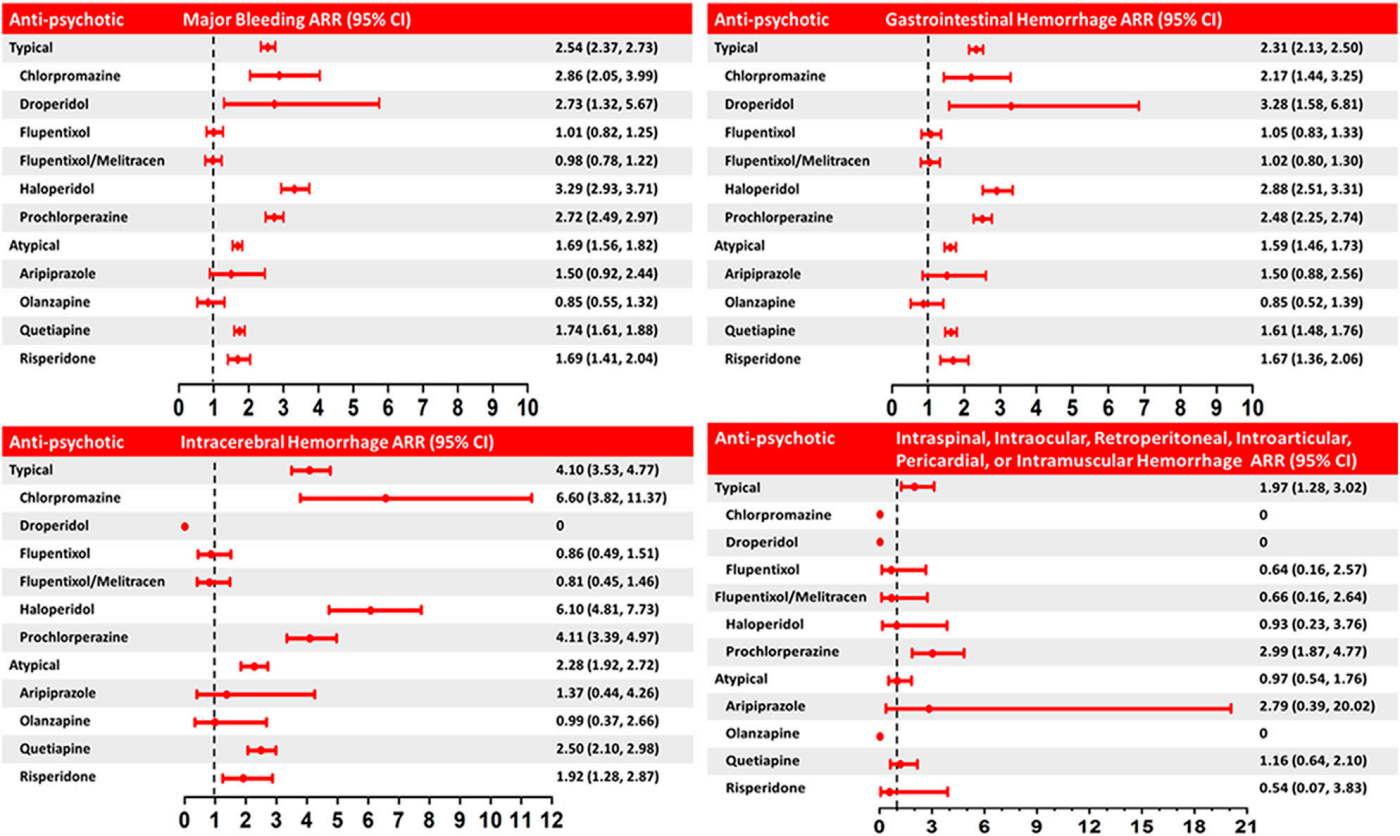
Adjusted rate ratio (ARR) of major bleeding and intracerebral, gastrointestinal, intraspinal, intraocular, retroperitoneal, intra-articular, pericardial, or intramuscular hemorrhage associated with the concurrent use of NOACs and anti-psychotics (typical and atypical) versus use of NOACs alone. The rate ratio was adjusted by inverse probability of treatment weighting using the propensity score (covariates including demographics, comorbidities, medications, and expense of medical utilization, in Tables 1, 2 and Supplementary Table S3). Horizontal lines represent 95% confidence intervals (CI).

Among NOAC users, 6,297 (6.37%) patients had one major bleeding event, and 782 (0.80%) patients had multiple major bleeding events. During the follow-up, 8,037 (1.14%) major bleeding events occurred during 705,521 person-quarters with NOAC prescriptions. In total, 66,342 (9.4%) person-quarters were prescribed antipsychotics. Multiple antipsychotics were prescribed in 1,121 (0.16%) person-quarters. Table 3 summarizes the incidence rate, adjusted incidence rate, adjusted incidence rate difference (AIRD), and adjusted rate ratio (ARR) (Figure 2) for major bleeding events, among the combinations of four NOACs and 10 antipsychotic use. The concomitant use of an NOAC with a typical (AIRD: 79.18, 95% confidence interval [CI]: 70.63–87.72, and *p* < 0.001) or an atypical (AIRD: 40.5, 95% CI: 33.64–47.35, and *p* < 0.001) antipsychotic showed a higher adjusted incidence rate and ARR of major bleeding than NOAC use alone. In individual drug analysis, the combination of an NOAC with chlorpromazine (AIRD: 103.87, 95% CI: 51.22–156.52, *p* < 0.001), haloperidol (AIRD: 149.52, 95% CI: 125.03–174.00, *p* < 0.001), prochlorperazine (AIRD: 90.43, 95% CI: 78.55–102.32, and *p* < 0.001), quetiapine (AIRD: 44.6, 95% CI: 37.11–52.09, and *p* < 0.001), or risperidone (AIRD: 41.55, 95% CI: 22.86–60.24, and *p* < 0.001) was associated with a higher adjusted incidence rate and ARR of major bleeding than NOAC alone. The combination of an NOAC and droperidol, aripiprazole, flupentixol, flupentixol/melitracen, or olanzapine was not associated with increased major bleeding events. Subgroup analysis showed a higher risk of intracerebral hemorrhage associated with the combination of an NOAC with a typical (AIRD: 21.69, 95% CI: 17.79–25.60, *p* < 0.001) or an atypical (AIRD: 12.15, 95% CI: 9.00–15.29, *p* < 0.001) antipsychotic compared with NOAC use alone (Table 4 and Figure 2). Individual drug analysis showed that the combination of chlorpromazine, haloperidol, prochlorperazine, quetiapine, or risperidone with NOAC use was associated with an increased risk of intracerebral bleeding compared with NOAC alone (Table 4 and Figure 2). Consistent with the results of intracerebral bleeding, the combination of NOACs with typical (AIRD: 55.77, 95% CI: 48.31–63.22, and *p* < 0.001) or atypical (AIRD: 28.3, 95% CI: 22.28–34.32, and *p* < 0.001) antipsychotics increased the adjusted incidence rate and ARR of gastrointestinal bleeding (Table 5 and Figure 2). Individual drug analysis showed that chlorpromazine, haloperidol, prochlorperazine, quetiapine, and risperidone, individually, increased the risk of gastrointestinal bleeding compared to NOAC use alone (Table 5 and Figure 2). The adjusted incidence rate and ARR of intraspinal, intraocular, retroperitoneal, intraarticular, pericardial, or intramuscular bleeding among AF patients were increased by the combination of NOACs with typical antipsychotics, as well as prochlorperazine (Supplementary Table S4 and Figure 2). Separate analyses showed that the concurrent use of antipsychotics with dabigatran, rivaroxaban, or apixaban, respectively, was similar to the overall pattern of bleeding risks demonstrated (Supplementary Tables S5–S7). However, the concurrent use of edoxaban with antipsychotics showed a different pattern from that of other NOACs used with concomitant antipsychotics (Supplementary Table S8).

**TABLE 3:** Major bleeding risks among AF patients taking non-vitamin K antagonist oral anticoagulants with or without concurrent antipsychotics.

Concurrent medication	Person-quarters with NOAC use	No. of bleeding events	Crude major bleeding incidence rate (95% CI) per 1000 person-years		Adjusted incidence rate (95% CI) per 1000 person-years^a^		Adjusted rate ratio (95% CI)^a^		Adjusted incidence rate difference (95% CI) per 1000 person-years	
Typical										
with	29,858	965	128.09	(119.92 to 136.82)	130.52	(122.33 to 139.26)	2.54^b^	(2.37 to 2.73)	79.18^b^	(70.63 to 87.72)
without^c^	675,663	7,072	42.35	(41.28 to 43.44)	51.34	(49.86 to 52.87)	1	(1.00 to 1.00)	—	—
Chlorpromazine										
with	963	38	153.28	(108.84 to 215.87)	159.61	(114.79 to 221.93)	2.86^b^	(2.05 to 3.99)	103.87^b^	(51.22 to 156.52)
without^c^	704,558	7,999	45.89	(44.80 to 47.02)	55.74	(53.27 to 58.32)	1	(1.00 to 1.00)	—	—
Droperidol										
with	192	7	143.03	(67.64 to 302.43)	146.08	(70.50 to 302.66)	2.73^b^	(1.32 to 5.67)	92.63	(–13.81 to 199.07)
without^c^	705,329	8,030	46.02	(44.92 to 47.15)	53.45	(51.45 to 55.52)	1	(1.00 to 1.00)	—	—
Flupentixol										
with	8,034	98	49.12	(39.72 to 60.75)	49.35	(39.93 to 61.01)	1.01	(0.82 to 1.25)	0.53	(−10.01 to 11.08)
without^c^	697,487	7,939	46.01	(44.91 to 47.14)	48.82	(47.30 to 50.39)	1	(1.00 to 1.00)	—	—
Flupentixol/Melitracen										
with	7,804	92	47.39	(38.00 to 59.09)	47.68	(38.26 to 59.41)	0.98	(0.78 to 1.22)	−1.18	(−11.75 to 9.39)
without^c^	697,717	7,945	46.03	(44.93 to 47.16)	48.85	(47.32 to 50.44)	1	(1.00 to 1.00)	—	—
Haloperidol										
with	5,613	299	208.06	(185.03 to 233.95)	214.71	(191.64 to 240.57)	3.29^b^	(2.93 to 3.71)	149.52^b^	(125.03 to 174.00)
without^c^	699908	7738	44.7	(43.62 to 45.82)	65.19	(62.99 to 67.48)	1	(1.00 to 1.00)	—	—
Prochlorperazine										
with	15,968	570	140	(128.67 to 152.32)	143.03	(131.66 to 155.37)	2.72^b^	(2.49 to 2.97)	90.43^b^	(78.55 to 102.32)
without^c^	689,553	7,467	43.81	(42.73 to 44.92)	52.59	(51.12 to 54.11)	1	(1.00 to 1.00)	—	—
Atypical										
with	41,497	1,017	97.97	(91.71 to 104.66)	99.59	(93.31 to 106.29)	1.69^b^	(1.56 to 1.82)	40.5^b^	(33.64 to 47.35)
without^c^	664,024	7,020	42.76	(41.67 to 43.88)	59.09	(56.76 to 61.52)	1	(1.00 to 1.00)	—	—
Aripiprazole										
with	874	19	85.08	(51.82 to 139.69)	86.63	(53.40 to 140.55)	1.5	(0.92 to 2.44)	28.9	(−13.13 to 70.92)
without^c^	704,647	8,018	46	(44.90 to 47.12)	57.74	(54.64 to 61.01)	1	(1.00 to 1.00)	—	—
Olanzapine										
with	1,662	20	47.36	(30.17 to 74.35)	48.47	(31.32 to 75.01)	0.85	(0.55 to 1.32)	−8.32	(−29.59 to 12.94)
without^c^	703,859	8,017	46.05	(44.95 to 47.17)	56.79	(54.72 to 58.95)	1	(1.00 to 1.00)	—	—
Quetiapine										
with	35,483	913	102.77	(95.88 to 110.17)	104.64	(97.72 to 112.05)	1.74^b^	(1.61 to 1.88)	44.6^b^	(37.11 to 52.09)
without^c^	670,038	7,124	43	(41.92 to 44.11)	60.04	(57.70 to 62.47)	1	(1.00 to 1.00)	—	—
Risperidone										
with	5,404	135	99.13	(82.23 to 119.51)	101.48	(84.50 to 121.88)	1.69^b^	(1.41 to 2.04)	41.55^b^	(22.86 to 60.24)
without^c^	700,117	7,902	45.63	(44.53 to 46.75)	59.93	(57.89 to 62.05)	1	(1.00 to 1.00)	—	—

a Adjusted by inverse probability of treatment weighting using the propensity score (gender, age, medical utilization, hypertension, myocardial infarction, congestive heart failure, percutaneous coronary intervention, coronary bypass surgery, peripheral vascular disease, cerebrovascular disease, ischemic stroke, transient ischemic attack, hemiplegia or paraplegia, dementia, epilepsy, diabetes mellitus, chronic kidney disease, chronic pulmonary disease, peptic ulcer disease, liver disease, malignancy, anemia, rheumatic disease, human immunodeficiency virus infection, antibiotics and anti-fungal drugs, anti-epileptics, anti-hypertensives, anti-platelets, bisphosphate, cardiovascular drugs, cyclosporine, glucocorticoid, insulin, lipid-lowering drugs, nonsteroid anti-inflammatory drugs, proton pump inhibitors, residence, income level, and occupation, *see* Tables 1, 2 and Supplementary Table S3).

b *p* < 0.05, compared with NOAC alone.

c Without indicates NOAC alone.

**TABLE 4:** Risk of intracerebral hemorrhage among AF patients taking non-vitamin K antagonist oral anticoagulants with or without concurrent antipsychotics.

Concurrent medication	Person-quarters with NOAC use	No. of bleeding events	Crude major bleeding incidence rate (95% CI) per 1,000 person-years		Adjusted incidence rate (95% CI) per 1,000 person-years^a^		Adjusted rate ratio (95% CI)^a^		Adjusted incidence rate difference (95% CI) per 1,000 person-years	
Typical										
with	29,858	212	28.61	(24.98 to 32.77)	28.68	(25.05 to 32.84)	4.1^b^	(3.53 to 4.77)	21.69^b^	(17.79 to 25.60)
without^c^	675,663	1,052	6.3	(5.92 to 6.71)	6.99	(6.52 to 7.50)	1	(1.00 to 1.00)	—	—
Chlorpromazine										
with	963	15	61.55	(35.52 to 106.64)	63.18	(36.91 to 108.14)	6.6^b^	(3.82 to 11.37)	53.6^b^	(19.64 to 87.55)
without^c^	704,558	1,249	7.17	(6.77 to 7.60)	9.58	(8.68 to 10.57)	1	(1.00 to 1.00)	—	—
Droperidol										
with	192	0	—	—	—	—	0	—	—	—
without^c^	705,329	1,264	—	—	—	—	1	(1.00 to 1.00)	—	—
Flupentixol										
with	8,034	12	6.1	(3.48 to 10.70)	6.01	(3.42 to 10.57)	0.86	(0.49 to 1.51)	−1	(−4.43 to 2.43)
without^c^	697,487	1,252	7.26	(6.85 to 7.69)	7.02	(6.55 to 7.52)	1	(1.00 to 1.00)	—	—
Flupentixol/Melitracen										
with	7,804	11	5.74	(3.19 to 10.33)	5.67	(3.15 to 10.22)	0.81	(0.45 to 1.46)	−1.35	(−4.72 to 2.03)
without^c^	697,717	1,253	7.26	(6.86 to 7.70)	7.02	(6.54 to 7.52)	1	(1.00 to 1.00)	—	—
Haloperidol										
with	5,613	78	55.4	(44.28 to 69.31)	56.04	(44.88 to 69.96)	6.1^b^	(4.81 to 7.73)	46.85^b^	(34.39 to 59.30)
without^c^	699,908	1,186	6.86	(6.46 to 7.28)	9.19	(8.43 to 10.01)	1	(1.00 to 1.00)	—	—
Prochlorperazine										
with	15,968	118	29.62	(24.73 to 35.48)	29.68	(24.79 to 35.54)	4.11^b^	(3.39 to 4.97)	22.45^b^	(17.09 to 27.81)
without^c^	689,553	1,146	6.73	(6.33 to 7.14)	7.23	(6.76 to 7.73)	1	(1.00 to 1.00)	—	—
Atypical										
with	41,497	219	21.19	(18.43 to 24.36)	21.61	(18.82 to 24.82)	2.28^b^	(1.92 to 2.72)	12.15^b^	(9.00 to 15.29)
without^c^	664,024	1,045	6.37	(5.98 to 6.78)	9.47	(8.49 to 10.55)	1	(1.00 to 1.00)	—	—
Aripiprazole										
with	874	3	13.37	(4.33 to 41.23)	13.46	(4.37 to 41.42)	1.37	(0.44 to 4.26)	—	—
without^c^	704,647	1,261	7.24	(6.84 to 7.67)	9.86	(8.18 to 11.88)	1	(1.00 to 1.00)	3.6	(−11.63 to 18.84)
Olanzapine										
with	1,662	4	9.67	(3.54 to 26.40)	9.89	(3.69 to 26.49)	0.99	(0.37 to 2.66)	—	—
without^c^	703,859	1,260	7.24	(6.84 to 7.67)	10.03	(9.01 to 11.16)	1	(1.00 to 1.00)	−0.13	(−9.94 to 9.67)
Quetiapine										
with	35,483	204	23.07	(19.96 to 26.66)	23.53	(20.38 to 27.16)	2.5^b^	(2.10 to 2.98)	14.12^b^	(10.62 to 17.63)
without^c^	670,038	1,060	6.4	(6.01 to 6.81)	9.4	(8.47 to 10.44)	1	(1.00 to 1.00)	—	—
Risperidone										
with	5,404	25	18.48	(12.37 to 27.60)	18.9	(12.76 to 28.01)	1.92^b^	(1.28 to 2.87)	9.05^b^	(1.57 to 16.53)
^c^without	700,117	1,239	7.16	(6.76 to 7.59)	9.86	(9.01 to 10.78)	1	(1.00 to 1.00)	—	—

a Adjusted by the inverse probability of treatment weighting using the propensity score (gender, age, medical utilization, hypertension, myocardial infarction, congestive heart failure, percutaneous coronary intervention, coronary bypass surgery, peripheral vascular disease, cerebrovascular disease, ischemic stroke, transient ischemic attack, hemiplegia or paraplegia, dementia, epilepsy, diabetes mellitus, chronic kidney disease, chronic pulmonary disease, peptic ulcer disease, liver disease, malignancy, anemia, rheumatic disease, human immunodeficiency virus infection, antibiotics and anti-fungal drugs, anti-epileptics, anti-hypertensives, anti-platelets, bisphosphate, cardiovascular drugs, cyclosporine, glucocorticoid, insulin, lipid-lowering drugs, nonsteroid anti-inflammatory drugs, proton pump inhibitors, residence, income level, and occupation, *see* Tables 1, 2 and Supplementary Table S3).

b *p* < 0.05, compared with NOAC alone.

c Without indicates NOAC alone.

**TABLE 5:** Risk of gastrointestinal hemorrhage among AF patients taking non-vitamin-K antagonist oral anticoagulants with or without concurrent antipsychotics.

Concurrent medication	Person- quarters with NOAC use	No. of bleeding events	Crude major bleeding incidence rate (95% CI) per 1,000 person-years		Adjusted incidence rate (95% CI) per 1,000 person-years^a^		Adjusted rate ratio (95% CI)^a^		Adjusted incidence rate difference (95% CI) per 1,000 person-years	
Typical										
with	29,858	730	96.22	(89.14 to 103.87)	98.49	(91.38 to 106.15)	2.31^b^	(2.13 to 2.50)	55.77^b^	(48.31 to 63.22)
without^c^	675,663	5,760	34.46	(33.49 to 35.46)	42.72	(41.34 to 44.15)	1	(1.00 to 1.00)	—	—
Chlorpromazine										
with	963	23	91.94	(60.32 to 140.13)	96.05	(64.19 to 143.71)	2.17^b^	(1.44 to 3.25)	51.71^b^	(12.94 to 90.49)
without^c^	704,558	6,467	37.06	(36.06 to 38.08)	44.34	(42.08 to 46.71)	1	(1.00 to 1.00)	—	—
Droperidol										
with	192	7	143.31	(67.87 to 302.64)	146.11	(70.52 to 302.71)	3.28^b^	(1.58 to 6.81)	101.59	(−4.87 to 208.05)
without^c^	705,329	6,483	37.1	(36.11 to 38.12)	44.52	(42.63 to 46.49)	1	(1.00 to 1.00)	—	—
Flupentixol										
with	8,034	84	41.86	(33.13 to 52.90)	42.23	(33.46 to 53.30)	1.05	(0.83 to 1.33)	2.07	(−7.83 to 11.98)
without^c^	697,487	6,406	37.08	(36.08 to 38.10)	40.16	(38.73 to 41.64)	1	(1.00 to 1.00)	—	—
Flupentixol/Melitracen										
with	7,804	79	40.46	(31.73 to 51.60)	40.88	(32.11 to 52.05)	1.02	(0.80 to 1.30)	0.69	(−9.26 to 10.64)
without^c^	697,717	6,411	37.1	(36.10 to 38.12)	40.19	(38.75 to 41.67)	1	(1.00 to 1.00)	—	—
Haloperidol										
with	5,613	219	151.31	(131.70 to 173.84)	157.08	(137.43 to 179.54)	2.88^b^	(2.51 to 3.31)	102.63^b^	(81.57 to 123.69)
without^c^	699,908	6,271	36.18	(35.20 to 37.19)	54.45	(52.40 to 56.58)	1	(1.00 to 1.00)	—	—
Prochlorperazine										
with	15,968	433	105.53	(95.69 to 116.37)	108.41	(98.53 to 119.28)	2.48^b^	(2.25 to 2.74)	64.73^b^	(54.34 to 75.13)
without^c^	689,553	6,057	35.5	(34.52 to 36.51)	43.68	(42.30 to 45.09)	1	(1.00 to 1.00)	—	—
Atypical										
with	41,497	784	75.34	(69.87 to 81.23)	76.53	(71.05 to 82.43)	1.59^b^	(1.46 to 1.73)	28.3^b^	(22.28 to 34.32)
without^c^	664,024	5,706	34.71	(33.73 to 35.73)	48.22	(46.13 to 50.41)	1	(1.00 to 1.00)	—	—
Aripiprazole										
with	874	15	67.01	(38.83 to 115.64)	68.85	(40.56 to 116.88)	1.5	(0.88 to 2.56)	23.04	(−13.47 to 59.54)
without^c^	704,647	6,475	37.1	(36.10 to 38.12)	45.81	(43.43 to 48.34)	1	(1.00 to 1.00)	—	—
Olanzapine										
with	1,662	16	37.67	(22.80 to 62.22)	38.61	(23.74 to 62.80)	0.85	(0.52 to 1.39)	−6.72	(−25.59 to 12.14)
without^c^	703,859	6,474	37.13	(36.14 to 38.15)	45.34	(43.55 to 47.20)	1	(1.00 to 1.00)	—	—
Quetiapine										
with	35,483	695	78.03	(72.06 to 84.50)	79.41	(73.42 to 85.89)	1.61^b^	(1.48 to 1.76)	30.15^b^	(23.62 to 36.69)
without^c^	670,038	5,795	34.94	(33.95 to 35.95)	49.26	(47.15 to 51.47)	1	(1.00 to 1.00)	—	—
Risperidone										
with	5,404	109	79.72	(64.68 to 98.25)	81.51	(66.40 to 100.07)	1.67^b^	(1.36 to 2.06)	32.82^b^	(16.02 to 49.62)
without^c^	700,117	6,381	36.8	(35.81 to 37.82)	48.69	(46.87 to 50.59)	1	(1.00 to 1.00)	—	—

a Adjusted by the inverse probability of treatment weighting using the propensity score (gender, age, medical utilization, hypertension, myocardial infarction, congestive heart failure, percutaneous coronary intervention, coronary bypass surgery, peripheral vascular disease, cerebrovascular disease, ischemic stroke, transient ischemic attack, hemiplegia or paraplegia, dementia, epilepsy, diabetes mellitus, chronic kidney disease, chronic pulmonary disease, peptic ulcer disease, liver disease, malignancy, anemia, rheumatic disease, human immunodeficiency virus infection, antibiotics and anti-fungal drugs, anti-epileptics, anti-hypertensives, anti-platelets, bisphosphate, cardiovascular drugs, cyclosporine, glucocorticoid, insulin, lipid-lowering drugs, nonsteroid anti-inflammatory drugs, proton pump inhibitors, residence, income level, and occupation, *see* Tables 1, 2 and Supplementary Table S3).

b *p* < 0.05, compared with NOAC alone.

c Without indicates NOAC alone.

The first sensitivity analysis examined the associations between NOACs with and without the concomitant use of an antipsychotic and appendicitis (as a negative control for a risk). None of the combinations was associated with a higher risk of appendicitis (Supplementary Table S9). The second sensitivity analysis evaluated whether including bleeding events that occurred within the first person-quarter of the concurrent NOAC and antipsychotic use would affect the main results, and with this analysis, we found the results were similar to the main findings (Supplementary Table S10).

## Discussion

This nationwide cohort study presents two main findings. First, antipsychotics were used in 26.35% of NOAC-exposed patients. Second, the concomitant use of either typical or atypical antipsychotics was associated with a significantly increased risk of major bleeding, including intracerebral and gastrointestinal hemorrhage, whereas the combined use of NOACs with typical and atypical antipsychotics increased the bleeding risk at other sites. While the coadministration of haloperidol, prochlorperazine, chlorpromazine, quetiapine, or risperidone significantly increased risks of major bleeding and intracerebral and gastrointestinal hemorrhage, only prochlorperazine was associated with a higher bleeding risk at other sites than NOAC use alone.

AF increases risks of dementia (Diener et al., 2019) and stroke (Zimetbaum, 2017), and psychotic disorders are commonly seen in both conditions (Kales et al., 2015; Stangeland et al., 2018). Antipsychotics are frequently prescribed to patients with dementia and stroke for controlling psychotic symptoms (Gareri et al., 2014; Kirkham et al., 2017). However, the frequency of antipsychotics used in NOAC users has never been reported. Our results showed 38.04 and 11.56% of NOAC users having ischemic stroke and dementia, respectively, which may explain the high frequency (26.35%) of antipsychotics used in NOAC-exposed patients in this study.

A previous case-control study has shown that antipsychotic use alone (including phenothiazine and butyrophenones) was associated with increased risks of gastrointestinal bleeding and intracerebral hemorrhage (Verdel et al., 2011). To our knowledge, this is the first piece of clinical evidence of increased bleeding risk of the concurrent use of antipsychotics in NOAC users in the context of a nationwide cohort. Previous cohort studies have shown associations of increased major bleeding risk with the concurrent use of NOACs and other medications including anti-epileptics, anti-arrhythmic, antifungals, and antibiotics in patients with non-valvular AF (Chang et al., 2017; Wang et al., 2020). In addition, previous review articles also reported clinical evidence of DDIs of NOACs with anti-inflammatory drugs, antivirals, antidepressants, immunosuppressants, and proton-pump inhibitors, but most of them are from cases reports (Stöllberger and Finsterer, 2015; Voukalis et al., 2016; Ingrasciotta et al., 2018).

Separate analyses showed the concurrent use of antipsychotics with dabigatran, rivaroxaban, or apixaban, respectively, was associated with a higher major bleeding risk than NOACs alone, similar to the overall pattern of bleeding risks demonstrated (Supplementary Tables S5–S7). The concurrent use of edoxaban with antipsychotics showed a different pattern from that of other NOACs used with antipsychotics (Supplementary Table S8). Co-medication of typical antipsychotics with edoxaban was not associated with an increased risk of major bleeding, whereas atypical antipsychotics with edoxaban increased the risk of major bleeding, particularly gastrointestinal hemorrhage. The discrepant results may arise from having fewer users of edoxaban than each of the other three NOACs and other unknown factors that remain to be examined further.

Among all the antipsychotics, the co-medication of haloperidol and NOACs is associated with the highest major bleeding risk. Haloperidol is primarily metabolized by CYP3A4, although it is also metabolized by CYP2D6, and studies have shown CYP3A4 plays a major role in drug interactions of haloperidol (Kudo and Ishizaki, 1999). Haloperidol is not only a substrate and inhibitor of CYP3A4 but also a P-gp inhibitor (El Ela et al., 2004; Iwaki et al., 2006; Lund et al., 2017). A comprehensive analysis of the clinical DDI studies has revealed that the impact of DDI mediated solely by P-gp inhibition may not be remarkably high, but the dual P-gp and CYP3A4 inhibition imposes a potent DDI risk (Umeyama et al., 2014).

Co-medication of chlorpromazine with NOACs was associated with the second highest major bleeding risk among all the antipsychotics but is associated with the highest risk of intracerebral hemorrhage. Chlorpromazine is a potent P-gp inhibitor, as demonstrated by an *in vitro* study (Wang et al., 2006). Chlorpromazine is mainly metabolized by CYP2D6 (Yoshii et al., 2000) and has been shown to be a modest CYP3A inhibitor by an *in vitro* study, but no effect on the CYP3A activity was demonstrated in an animal study (Wójcikowski et al., 2012). It is not known how much of the results of *in vitro* and *in vivo* studies can be translated to activity in humans. Nevertheless, the present study suggests a significantly increased DDI risk of chlorpromazine and NOACs in clinical practice.

Prochlorperazine was the most frequently prescribed antipsychotic in NOAC users. Prochlorperazine is extensively metabolized by CYP2C19, CYP2D6, and CYP3A4/5 (Finn et al., 2005). In addition to its antipsychotic effect, prochlorperazine has been used as an emetic for many years and is considered to be an effective multiple drug resistance-reverser in cancer treatment due to its potent P-gp inhibition activity (Pajak et al., 2005). Prochlorperazine is the only antipsychotic that increased the risks of all kinds of major bleeding when concurrently taken with NOACs versus the use of NOACs alone.

Among atypical antipsychotics, quetiapine, and risperidone have similar major bleeding rate ratios, whereas quetiapine has a higher intracranial hemorrhage ARR than that of risperidone, which may be attributed to the feature of quetiapine being a stronger P-gp inhibitor than risperidone (Tsai et al., 2020). In addition, quetiapine is mainly metabolized by CYP3A4 (DeVane and Nemeroff, 2001), whereas risperidone is metabolized by both CYP3A4 and CYP2D6 (Bork et al., 1999), which may explain the higher major bleeding risk of quetiapine than risperidone when co-medicated with a NOAC.

Aripiprazole is substantially metabolized by CYP2D6 and CYP3A4 (Citrome et al., 2005). No pharmacokinetic interaction was shown in the concurrent use of aripiprazole and valproic acid, a CYP3A4 inhibitor (Citrome et al., 2005), suggesting the role of CYP3A4 in the metabolism of aripiprazole may be less significant. However, aripiprazole is a substrate of P-gp in a P-gp–deficient animal model (Kirschbaum et al., 2010). Despite the previous studies, our study reveals clinical evidence that the DDI risk of aripiprazole and NOACs may be minor. Studies have shown olanzapine is metabolized primarily by the CYP1A2 and direct glucuronidation (Urichuk et al., 2008). Given that CYP3A4 plays a very minor role in the metabolism of olanzapine, this minor role may explain why olanzapine did not increase the major bleeding risk in NOAC users.

Atypical antipsychotics are being prescribed preferentially, due to having fewer adverse effects of extrapyramidal symptoms, than typical antipsychotics. Here, our study demonstrates that atypical anti-psychotics also had a less increased bleeding risk than typical ones when combined with NOAC use. It is noted that atypical antipsychotics increased the occurrence of the metabolic syndrome (Pramyothin and Khaodhiar, 2010; Piccinni et al., 2020), and both typical and atypical antipsychotics are significantly associated the with increased risk of stroke and ischemic heart events (Zivkovic et al., 2019; Piccinni et al., 2020). Since both typical and atypical antipsychotics may increase thromboembolic stroke and a major bleeding risk due to DDIs with NOACs, it is highly recommended to avoid prescribing the antipsychotics associated with increased bleeding risks in NOAC users. When it is necessary to prescribe antipsychotics for NOAC users, atypical antipsychotics such as olanzapine and aripiprazole may be considered. Furthermore, a previous study has shown that a campaign for general practitioners to reduce DDIs in polypharmacy elderly patients significantly decreases most prevalent DDIs, especially the NSAID-related DDIs (Raschi et al., 2015). The present study addressed the need to include NOACs and antipsychotics among specific lists of clinically important DDIs.

### Study Strengths

Our study has several strengths. First, this is a nationwide cohort study, which has taken into account a wide range of covariates including co-morbidities and other medicines that may affect bleeding risks. Second, we used the propensity score weighting to account for nonrandom assignment differences between NOAC users with and without concurrent medication of antipsychotics. In the first sensitivity analysis, we used appendicitis as a negative control outcome to exclude the unobserved confounding effect. In the second sensitivity analysis, bleeding events occurring within the first person-quarter of comedication were not included to exclude the bleeding events that were caused probably not by a newly added antipsychotic but other existing medications or factors. Third, up to now, no clinical or *in vitro* and *in vivo* pharmacokinetic data regarding DDIs of antipsychotics with NOACs have been reported. Our study is the first to address some parts of the important but unanswered questions.

### Study Limitations

There are limitations to this study. First, the bias caused by unmeasured confounding factors could not be completely corrected by propensity score weighting. For example, the liver and renal function data were not available in the NHI database, and the dosage, duration, and compliance of NOACs and antipsychotics were not included in this study, which leaves it unclear if the increased bleeding risks are influenced by these factors. Second, misclassification or ICD miscoding in the nationwide cohort studies has become a concern, which could lead to biased results, although large data sets may minimize the bias. Third, the edoxaban users were fewer than the other three NOACs, which raises a question about extrapolation of the results regarding co-medication of antipsychotics and edoxaban to the general population. A similar issue could occur when interpreting the results of the concurrent use of NOACs and olanzapine or aripiprazole. Fourth, although many confounding factors have been taken into account, drug–food interactions were not incorporated into the analysis. Fifth, since patients diagnosed with pulmonary embolism, deep vein thrombosis, mitral stenosis, end-stage renal disease, joint replacement, or valvular surgery with a higher risk of bleeding were excluded in this study, our study results cannot be generalized to these patients. Furthermore, this study did not examine individual characteristics of pharmacokinetics (CES1, CYP3A4, CYP3A5, CYP2J9, CYP2D6, and CYP2C19 gene polymorphisms, etc.), which may determine individual characteristics of the pharmacokinetics of both NOACs and antipsychotics and how DDIs of both drugs influence the risk of bleeding. Last, the ethnic differences in the bleeding risk and efficacy of NOACs and the epidemiology and outcomes of AF may limit the application of our study results to other ethnic populations (Nanda and Kabra, 2019).

## Conclusion

Antipsychotics are frequently prescribed to non-valvular AF patients taking NOACs in a national cohort. Among those NOAC users, the concurrent use of haloperidol, prochlorperazine, chlorpromazine, quetiapine, or risperidone significantly increased the risks of major bleeding. The data demonstrated the clinical evidence of increased bleeding risks potentially resulting from DDIs of antipsychotics and NOACs, which can lay a foundation for further studies of DDIs and help in developing a practical guide on the prescription of antipsychotics to NOAC users in the future.

## Data Availability

The original contributions presented in the study are included in the article/Supplementary Material, further inquiries can be directed to the corresponding author.
