# Supplementary figures and images for: Major Bleeding Risk in Atrial Fibrillation Patients Co-Medicated With Non-Vitamin K Oral Anticoagulants and Antipsychotics
Source: Front Pharmacol. 2022 Apr 14;13:819878. doi: 10.3389/fphar.2022.819878 (PMC9046567; doi:10.3389/fphar.2022.819878)

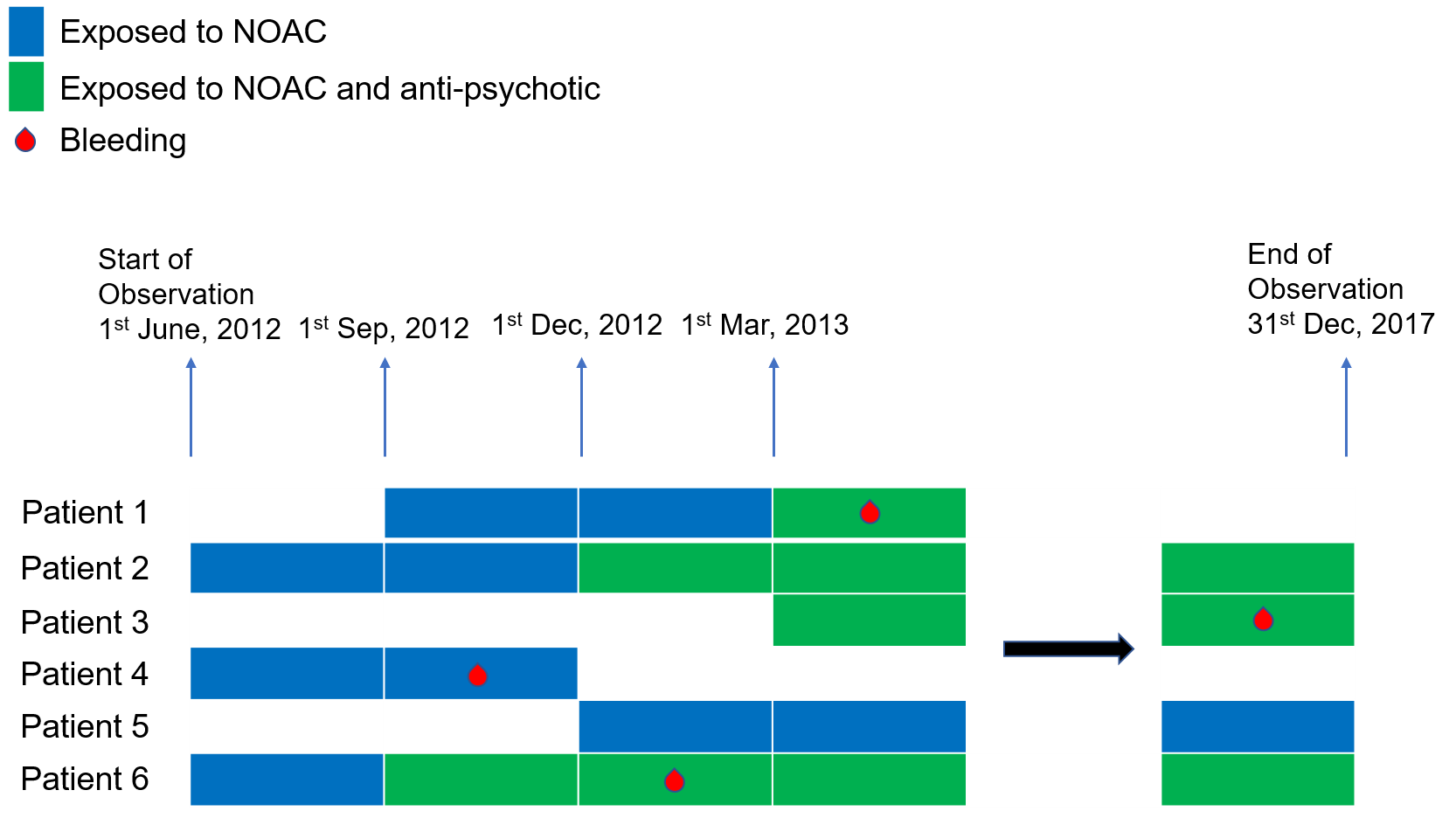

Supplement: Supplementary file 1 [file Image1.TIF]
