# Supplementary material for: Major Bleeding Risk in Atrial Fibrillation Patients Co-Medicated With Non-Vitamin K Oral Anticoagulants and Antipsychotics
Source: Front Pharmacol. 2022 Apr 14;13:819878. doi: 10.3389/fphar.2022.819878 (PMC9046567; doi:10.3389/fphar.2022.819878)
Supplement: Supplementary file 2 [file DataSheet1.docx]

**Table S1**. Definitions and codes of major bleeding and negative control outcomes.

|  | ICD-9-CM | | ICD-10 | |
| --- | --- | --- | --- | --- |
| Intracranial | 430, 431, 432.0, 432.1, 432.9 | | I60, I61, I62 | |
| Gastrointestinal | 530.7, 531, 531.2, 531.4, 531.6, 532, 532.2, 532.4, 532.6, 533, 533.2, 533.4, 533.6, 534, 534.2, 534.4, 534.6, 569.3, 535.01, 53.11, 535.21, 535.31, 535.41, 535.51, 535.61, 535.71, 537.71, 537.83, 537.84, 562.02, 562.03, 562.12, 562.13, 569.85, 578 | | K22.6, K25–K28, K29.01, K29.21, K29.31, K29.41, K29.51, K29.61, K29.71, K29.81, K29.91, K31.811, K31.82, K52.81, K55.21, K56.60, K57.01, K57.11, K57.13, K57.21, K57.31, K57.33, K57.81, K57.91, K57.93, K62.5, K92.0, K92.1, K92.2 | |
| Other sites (intraspinal, intraocular, retroperitoneal, intra-articular, pericardial, or intramuscular bleeding) | 336.1, 363.6, 372.72, 376.32, 377.42, 379.23, 593.81, 866.01, 866.02, 866.11, 866.12, 719.1, 729.92, 423.0, 772.5 | | G95.11, G95.19, H05.23, H11.3, H31.3, H43.1, H47.02, I31.2, M25.0, N28.0, P54.4, S31.001A, S37.011A, S37.012A, S37.019A, S37.021A, S37.022A, S37.029A, S37.031A, A37.032A, S37.039A, S37.041A, S37.042A, S37.049A, S37.051A, S37.052A, S37.059A | |
| Acute pancreatitis | | 577.0 | | K85 |
| Intestinal obstruction †without mention of hernia | | 560 | | K56 |
| Acute appendicitis | | 540, 541 | | K35 |

**Table S2.** Definitions and codes of atrial fibrillation and covariates.

|  | ICD-9-CM | ICD-10 |
| --- | --- | --- |
| Atrial fibrillation | 427.31 | I48.0, I48.2, I48.91 |
| Hypertension | 401, 402 | I10–I16 |
| Myocardial infarction | 410, 412 | I21, I22, I25.2 |
| Congestive heart failure | 398.91, 402.01, 402.11, 402.91, 404.01, 404.03, 404.11, 404.13, 404.91, 404.93, 425.4–425.9, 428 | I09.9, I11.0, I13.0, I13.2, I25.5, I42.0, I42.5–I42.9, I43, I50, I29.0 |
| Percutaneous coronary intervention* | 33076A, 33076B, 33077A, 33077B, 33078A, 33078B | |
| Coronary artery bypass surgery* | 68023A, 68023B, 68024A, 68024B, 68025A, 68025B | |
| Peripheral vascular disease | 093.0, 437.3, 440, 441, 443.1–443.9, 557.1, 557.9, V43.4 | I70, I71, I73.1, I73.8, I73.9, I77.1, I79.0, I79.2, K55.1, K55.8, K55.9, Z95.8, Z95.9 |
| Cerebrovascular disease | 362.34, 430–438 | G45, G46, H34.0, I60–I69 |
| Ischemic stroke | 433–434, 436, 852, 853 | I67.89, I63–I64, G458–G459, S01.90XA, S06.4X0A–S06.4X9A, S06.5X0A–S06.5X9A, S06.6X0A–S06.6X9A, S06.340A–S06.349A, S06.350A–S06.359A, S06.360A–S06.369A |
| Transient ischemic attack | 435 | G45 |
| Hemiplegia or paraplegia | 334.1, 342, 343, 344.0–344.6, 344.9 | G04.1, G11.4, G80.1, G80.2, G81, G82, G83.0–G83.4, G83.9 |
| Dementia | 290, 294.1, 331.2 | F00–F03, F05.1, G30, G31.1 |
| Epilepsy | 345 | G40 |
| Diabetes mellitus | 250 | E10.0, E10.1, E10.9, E11.0, E11.1, E11.9 |
| Diabetes with complications | 250.4–250.7 | E10.2–E10.5, E10.7, E11.2–E11.5, E11.7, E12.2–E12.5, E12.7, E13.2–E13.5, E13.7, E14.2–E14.5, E14.7 |
| Chronic kidney disease | 580-589 | I12, I13, N00–N05, N07, N11, N14, N17, N18, N19, Q61 |
| Chronic pulmonary disease | 416.8, 416.9, 490–505, 506.4, 508.1, 508.8 | I27.8, I27.9, J40–J47, J60–J67, J68.4, J70.1, J70.3 |
| Chronic obstructive pulmonary disease | 490, 491.0, 491.1, 491.20, 491.21, 491.22, 491.8, 492.0, 492.8, 494, 496 | J40, J41.0, J41.1, J41.8, J42, J43.0, J43.1, J43.2, J43.8, J43.9, J44.0, J44.1, J44.9, J47.0, J47.1, J47.9 |
| Peptic ulcer disease | 531–534 | K25–K28 |
| Mild liver disease | 070.22, 070.23, 070.32, 070.33, 070.44, 070.54, 070.6, 070.9, 570, 571, 573.3, 573.4, 573.8, 573.9 | B18, K70.0–K70.3, K70.9, K71.3–K71.5, K71.7, K73, K74, K76.0, K76.2–K76.4, K76.8, K76.9, Z94.4 |
| Moderate or severe liver disease | 456.0–456.2, 572.2–572.8 | I85.0, I85.9, I86.4, I98.2, K70.4, K71.1, K72.1, K72.9, K76.5, K76.6, K76.7 |
| Any malignancy, including lymphoma and leukemia, except malignant neoplasm of skin | 140–172, 174-195.8, 200–208, 238.6 | C00–C26, C30–C34, C37–C41, C43, C45–C58, C60–C76, C81–C85, C88, C90–C97 |
| Metastatic solid tumor | 196–199 | C77–C80 |
| Human immunodeficiency virus infection | 042–044 | B20–B22, B24 |
| Anemia | 280–285 | D46.1, D46.4, D50–D64 |
| Rheumatic disease | 446.5, 710.0–710.4, 714.0–714.2, 714.8, 725 | M05, M06, M31.5, M32–M34, M35.1, M35.3, M36.0 |

*Percutaneous coronary intervention / coronary artery bypass surgery is procedure code.

**Table S3.** Medications during follow up.

| Medications | NOACs users (n = 98863) |
| --- | --- |
| Antibiotics and antifungal drugs | 2800 (2.83%) |
| Erythromycin (%) | 2277 (2.3%) |
| Itraconazole (%) | 64 (0.06) |
| Ketoconazole (%) | 163 (0.16%) |
| Rifampin (%) | 311 (0.31%) |
| Anticoagulants | 98863 (100%) |
| Apixaban | 20825 (21.06%) |
| Dabigatran | 42779 (43.27%) |
| Edoxaban | 10517 (10.64%) |
| Rivaroxaban | 55587 (56.23%) |
| Antiepileptics | 4928 (4.98%) |
| Carbamazepine | 189 (0.19%) |
| Gabapentin | 758 (0.77%) |
| Lamotrigine | 42 (0.04%) |
| Levetiracetam | 1081 (1.09%) |
| Oxcarbazepine | 322 (0.33%) |
| Phenobarbital | 814 (0.82%) |
| Phenytoin | 651 (0.66%) |
| Pregabalin | 408 (0.41%) |
| Topiramate | 138 (0.14%) |
| Valproate | 1289 (1.3%) |
| Antihypertensives | 59 324 (60.1%) |
| Bisoprolol (%) | 33 396 (33.78%) |
| Diltiazem (%) | 15 284 (15.46%) |
| Irbesartan (%) | 4478 (4.53%) |
| Labetalol (%) | 1187 (1.2%) |
| Losartan (%) | 8784 (8.89%) |
| Metoprolol (%) | 1131 (1.14%) |
| Olmesartan (%) | 6055 (6.12%) |
| Propranolol (%) | 8026 (8.12%) |
| Antiplatelets | 24 186 (24.46%) |
| Aspirin (%) | 13 873 (14.03%) |
| Cilostazol | 1792 (1.81%) |
| Clopidogrel (%) | 6063 (6.13%) |
| Dipyridamole | 3404 (3.44%) |
| Pentoxifylline | 3724 (3.77%) |
| Ticagrelor (%) | 376 (0.38%) |
| Ticlopidine (%) | 808 (0.82%) |
| Anti-psychotics | 26041 (26.34%) |
| Typical |  |
| Prochlorperazine | 12159 (12.30%) |
| Haloperidol  Flupentixol  flupentixolM  Chlorpromazine  Droperidol | 4616 (4.67% )  3267 (3.30%)  3201 (3.23%)  685 (0.69%)  190 (0.19%) |
| Atypical |  |
| Quetiapine  Risperidone  Olanzapine  Aripiprazole | 11770 (11.90%)  2326 (2.35%)  751 (0.76%)  403 (0.41%) |
| Bisphosphate (%) | 602 (0.61%) |
| Cardiovascular drugs | 36 505 (36.92%) |
| Amiodarone (%) | 20 328 (20.56%) |
| Digoxin (%) | 16 933 (17.13%) |
| Dronedarone (%) | 2907 (2.94%) |
| Cyclosporine (%) | 49 (0.05%) |
| Glucocorticoid (%) | 7987 (8.08%) |
| Insulin (%) | 6236 (6.31%) |
| Lipid lowering drugs | 19 172 (19.39%) |
| Atorvastatin (%) | 12 654 (12.8%) |
| Ezetimibe (%) | 82 (0.08%) |
| Fluvastatin (%) | 1436 (1.45%) |
| Pravastatin (%) | 1600 (1.62%) |
| Pitavastatin (%) | 3726 (3.77%) |
| Non-steroid anti-inflammatory drugs (%) | 19 180 (19.4%) |

NOACs: non-vitamin K oral anticoagulants

**Table S4**. Risk of intraspinal, intraocular, retroperitoneal, intra-articular, pericardial, or intramuscular hemorrhage among AF patients taking non-vitamin-K antagonist oral anticoagulants with or without concurrent anti-psychotics.

| **Concurrent medication** | **Person-Quarters with NOAC use** | **No. of Bleeding Events** | **Crude Major Bleeding Incidence Rate (95% CI) per 1000 Person-Years** | | ***Adjusted Incidence Rate (95% CI) per 1000 Person-Years** | | ***Adjusted Rate Ratio (95% CI)** | | ***Adjusted Incidence Rate Difference (95% CI) per 1000 Person-Years** | |
| --- | --- | --- | --- | --- | --- | --- | --- | --- | --- | --- |
| Typical |  |  |  |  |  |  |  |  |  |  |
| with | 29858 | 23 | 3.08 | (2.05-4.64) | 3.08 | (2.05-4.64) | #1.97 | (1.28-3.02) | #1.51 | (0.24-2.79) |
| †without | 675663 | 260 | 1.54 | (1.36-1.74) | 1.57 | (1.37-1.79) | 1 | (1.00-1.00) |  |  |
| Chlorpromazine |  |  |  |  |  |  |  |  |  |  |
| with | 963 | 0 |  |  |  |  | 0 |  |  |  |
| †without | 704558 | 283 |  |  |  |  | 1 | (1.00-1.00) |  |  |
| Droperidol |  |  |  |  |  |  |  |  |  |  |
| with | 192 | 0 | 0 | (0.00-0.00) |  |  | 0 |  |  |  |
| †without | 705329 | 283 | 1.6 | (1.42-1.81) |  |  | 1 | (1.00-1.00) |  |  |
| Flupentixol |  |  |  |  |  |  |  |  |  |  |
| with | 8034 | 2 | 1 | (0.25-3.98) | 1 | (0.25-3.98) | 0.64 | (0.16-2.57) | -0.56 | (-1.96-0.83) |
| †without | 697487 | 281 | 1.61 | (1.43-1.82) | 1.56 | (1.35-1.80) | 1 | (1.00-1.00) |  |  |
| Flupentixol/Melitracen | |  |  |  |  |  |  |  |  |  |
| with | 7804 | 2 | 1.02 | (0.26-4.09) | 1.03 | (0.26-4.09) | 0.66 | (0.16-2.64) | -0.54 | (-1.98-0.90) |
| †without | 697717 | 281 | 1.61 | (1.43-1.82) | 1.56 | (1.36-1.80) | 1 | (1.00-1.00) |  |  |
| Haloperidol |  |  |  |  |  |  |  |  |  |  |
| with | 5613 | 2 | 1.43 | (0.36-5.70) | 1.43 | (0.36-5.70) | 0.93 | (0.23-3.76) | -0.1 | (-2.09-1.89) |
| †without | 699908 | 281 | 1.61 | (1.42-1.81) | 1.53 | (1.31-1.78) | 1 | (1.00-1.00) |  |  |
| Prochlorperazine |  |  |  |  |  |  |  |  |  |  |
| with | 15968 | 19 | 4.76 | (3.04-7.46) | 4.76 | (3.04-7.46) | #2.99 | (1.87-4.77) | #3.17 | (1.02-5.31) |
| †without | 689553 | 264 | 1.53 | (1.35-1.73) | 1.59 | (1.39-1.82) | 1 | (1.00-1.00) |  |  |
| Atypical |  |  |  |  |  |  |  |  |  |  |
| with | 41497 | 14 | 1.35 | (0.77-2.36) | 1.35 | (0.77-2.36) | 0.97 | (0.54-1.76) | -0.03 | (-0.84-0.77) |
| †without | 664024 | 269 | 1.62 | (1.43-1.83) | 1.38 | (1.14-1.68) | 1 | (1.00-1.00) |  |  |
| Aripiprazole |  |  |  |  |  |  |  |  |  |  |
| with | 874 | 1 | 4.57 | (0.64-32.59) | 4.58 | (0.64-32.60) | 2.79 | (0.39-20.02) | 2.94 | (-6.05-11.93) |
| †without | 704647 | 282 | 1.6 | (1.42-1.80) | 1.64 | (1.41-1.91) | 1 | (1.00-1.00) |  |  |
| Olanzapine |  |  |  |  |  |  |  |  |  |  |
| with | 1662 | 0 | 0 | (0.00-0.00) |  |  | 0 |  |  |  |
| †without | 703859 | 283 | 1.61 | (1.43-1.81) |  |  | 1 | (1.00-1.00) |  |  |
| Quetiapine |  |  |  |  |  |  |  |  |  |  |
| with | 35483 | 14 | 1.58 | (0.90-2.76) | 1.58 | (0.90-2.76) | 1.16 | (0.64-2.10) | 0.22 | (-0.70-1.14) |
| †without | 670038 | 269 | 1.61 | (1.42-1.81) | 1.36 | (1.12-1.65) | 1 | (1.00-1.00) |  |  |
| Risperidone |  |  |  |  |  |  |  |  |  |  |
| with | 5404 | 1 | 0.74 | (0.10-5.26) | 0.74 | (0.10-5.26) | 0.54 | (0.07-3.83) | -0.64 | (-2.11-0.83) |
| †without | 700117 | 282 | 1.61 | (1.43-1.82) | 1.38 | (1.17-1.64) | 1 | (1.00-1.00) |  |  |

*Adjusted by inverse probability of treatment weighting using the propensity score (gender, age, medical utilization, hypertension, myocardial infarction, congestive heart failure, percutaneous coronary intervention, coronary bypass surgery, peripheral vascular disease, cerebrovascular disease, ischemic stroke, transient ischemic attack, hemiplegia or paraplegia, dementia, epilepsy, diabetes mellitus, chronic kidney disease, chronic pulmonary disease, peptic ulcer disease, liver disease, malignancy, anemia, rheumatic disease, human immunodeficiency virus infection, anti-biotics and anti-fungal drugs, anti-epileptics, anti-hypertensives, anti-platelets, bisphosphate, cardiovascular drugs, cyclosporine, glucocorticoid, insulin, lipid lower drugs, nonsteroid anti–inflammatory drugs, proton pump inhibitors, residence, income level, and occupation, see **Tables 1 and 2, and Supplementary Table 3**).

†Without indicates NOAC alone.

#*P* < 0.05, compared with NOAC alone.

**Table S5**. Bleeding risk among AF patients taking dabigatran with or without concurrent anti-psychotics.

| **Concurrent medication** | **Person- Quarters with NOAC use** | **No. of Bleeding Events** | **Crude Major Bleeding Incidence Rate (95% CI) per 1000 Person-Years** | | ***Adjusted Incidence Rate (95% CI) per 1000 Person-Years** | | ***Adjusted Rate Ratio (95% CI)** | | ***Adjusted Incidence Rate Difference (95% CI) per 1000 Person-Years** | |
| --- | --- | --- | --- | --- | --- | --- | --- | --- | --- | --- |
| **Major bleeding** | |  |  |  |  |  |  |  |  |  |
| Typical |  |  |  |  |  |  |  |  |  |  |
| with | 11223 | 355 | 126.41 | (113.60-140.67) | 127.44 | (114.60-141.73) | #2.87 | (2.56-3.22) | #83.1 | (69.48-96.72) |
| †without | 259341 | 2362 | 36.89 | (35.34-38.51) | 44.34 | (42.24-46.54) | 1 | (1.00-1.00) |  |  |
| Atypical |  |  |  |  |  |  |  |  |  |  |
| with | 14368 | 334 | 93.43 | (83.50-104.54) | 94.27 | (84.34-105.38) | #1.74 | (1.52 -1.99) | #40.19 | (28.96-51.42) |
| †without | 256196 | 2383 | 37.69 | (36.10-39.35) | 54.09 | (50.19-58.29) | 1 | (1.00 -1.00) |  |  |
| **Intracerebral hemorrhage** | |  |  |  |  |  |  |  |  |  |
| Typical |  |  |  |  |  |  |  |  |  |  |
| with | 11223 | 70 | 25.01 | (19.78-31.64) | 25.09 | (19.85-31.72) | #4.48 | (3.44-5.85) | 19.5 | (13.59- 25.41) |
| †without | 259341 | 320 | 4.99 | (4.46-5.58) | 5.6 | (4.92-6.36) | 1 | (1.00-1.00) |  |  |
| Atypical |  |  |  |  |  |  |  |  |  |  |
| with | 14368 | 63 | 17.66 | (13.72-22.75) | 18.03 | (14.01-23.19) | #2.16 | (1.57-2.97) | #9.67 | (4.83-14.50) |
| †without | 256196 | 327 | 5.15 | (4.61-5.76) | 8.36 | (6.87-10.17) | 1 | (1.00-1.00) |  |  |
| **Gastrointestinal hemorrhage** | |  |  |  |  |  |  |  |  |  |
| Typical |  |  |  |  |  |  |  |  |  |  |
| with | 11223 | 276 | 98.09 | (86.85-110.79) | 98.96 | (87.69-111.69) | #2.65 | (2.33-3.02) | #61.64 | (49.59-73.69) |
| †without | 259341 | 1955 | 30.52 | (29.10-32.00) | 37.33 | (35.39-39.37) | 1 | (1.00-1.00) |  |  |
| Atypical |  |  |  |  |  |  |  |  |  |  |
| with | 14368 | 264 | 73.66 | (64.95-83.55) | 74.25 | (65.54-84.12) | #1.66 | (1.43-1.93) | #29.59 | (19.65-39.53) |
| †without | 256196 | 1967 | 31.09 | (29.64-32.61) | 44.66 | (41.13-48.49) | 1 | (1.00-1.00) |  |  |
| **Intraspinal, intraocular, retroperitoneal, intra-articular, pericardial, or intramuscular hemorrhage** | | | | | | | | |  |  |
| Typical |  |  |  |  |  |  |  |  |  |  |
| with | 11223 | 9 | 3.21 | (1.67-6.17) | 3.21 | (1.67-6.17) | #2.36 | (1.18-4.72) | 1.85 | (-0.27-3.97) |
| †without | 259341 | 87 | 1.34 | (1.08-1.66) | 1.36 | (1.08-1.71) | 1 | (1.00 -1.00) |  |  |
| Atypical |  |  |  |  |  |  |  |  |  |  |
| with | 14368 | 7 | 1.95 | (0.93-4.09) | 1.95 | (0.93-4.09) | 1.88 | (0.85-4.16) | 0.91 | (-0.56-2.39) |
| †without | 256196 | 89 | 1.39 | (1.12-1.72) | 1.04 | (0.78-1.38) | 1 | (1.00-1.00) |  |  |

*Adjusted by inverse probability of treatment weighting using the propensity score (gender, age, medical utilization, hypertension, myocardial infarction, congestive heart failure, percutaneous coronary intervention, coronary bypass surgery, peripheral vascular disease, cerebrovascular disease, ischemic stroke, transient ischemic attack, hemiplegia or paraplegia, dementia, epilepsy, diabetes mellitus, chronic kidney disease, chronic pulmonary disease, peptic ulcer disease, liver disease, malignancy, anemia, rheumatic disease, human immunodeficiency virus infection, anti-biotics and anti-fungal drugs, anti-epileptics, anti-hypertensives, anti-platelets, bisphosphate, cardiovascular drugs, cyclosporine, glucocorticoid, insulin, lipid lower drugs, nonsteroid anti–inflammatory drugs, proton pump inhibitors, residence, income level, and occupation, see **Tables 1 and 2, and Supplementary Table 3**).

†Without indicates NOAC alone.

#*P* < 0.05, compared with NOAC alone.

**Table S6**. Bleeding risk among AF patients taking rivaroxaban with or without concurrent anti-psychotics.

| **Concurrent medication** | **Person- Quarters with NOAC use** | **No. of Bleeding Events** | **Crude Major Bleeding Incidence Rate (95% CI) per 1000 Person~Years** | | ***Adjusted Incidence Rate (95% CI) per 1000 Person~Years** | | ***Adjusted Rate Ratio (95% CI)** | | ***Adjusted Incidence Rate Difference (95% CI) per 1000 Person-Years** | |
| --- | --- | --- | --- | --- | --- | --- | --- | --- | --- | --- |
| **Major bleeding** |  |  |  |  |  |  |  |  |  |  |
| Typical |  |  |  |  |  |  |  |  |  |  |
| with | 14986 | 469 | 124.49 | (113.26-136.82) | 126.47 | (115.24-138.80) | #2.26 | (2.05 -2.50) | #70.55 | (58.62- 82.47) |
| †without | 322706 | 3709 | 46.59 | (44.99-48.25) | 55.92 | (53.74-58.20) | 1 | (1.00 -1.00) |  |  |
| Atypical |  |  |  |  |  |  |  |  |  |  |
| with | 21217 | 533 | 100.86 | ( 92.20-110.35) | 102 | (93.31-111.50) | #1.62 | (1.46-1.80) | #39.06 | (29.41-48.71) |
| †without | 316475 | 3645 | 46.68 | ( 45.07- 48.35) | 62.94 | (59.57-66.51) | 1 | (1.00-1.00) |  |  |
| **Intracerebral hemorrhage** | |  |  |  |  |  |  |  |  |  |
| Typical |  |  |  |  |  |  |  |  |  |  |
| with | 14986 | 106 | 28.61 | (23.60-34.68) | 28.64 | (23.62-34.71) | #3.59 | (2.90-4.44) | #20.65 | (15.10-26.20) |
| †without | 322706 | 584 | 7.32 | (6.73-7.96) | 7.98 | (7.27-8.77) | 1 | (1.00-1.00) |  |  |
| Atypical |  |  |  |  |  |  |  |  |  |  |
| with | 21217 | 129 | 24.37 | (20.30-29.25) | 24.72 | (20.62-29.64) | #2.42 | (1.92-3.05) | #14.51 | (9.80-19.22) |
| †without | 316475 | 561 | 7.18 | (6.59-7.82) | 10.21 | (8.87-11.76) | 1 | (1.00-1.00) |  |  |
| **Gastrointestinal hemorrhage** | |  |  |  |  |  |  |  |  |  |
| Typical |  |  |  |  |  |  |  |  |  |  |
| with | 14986 | 349 | 91.9 | (82.29-102.64) | 93.77 | (84.16-104.49) | #2.04 | (1.82 -2.29) | #47.82 | (37.53-58.12) |
| †without | 322706 | 2974 | 37.34 | (35.90-38.84) | 45.95 | (43.93-48.07) | 1 | (1.00 -1.00) |  |  |
| Atypical |  |  |  |  |  |  |  |  |  |  |
| with | 21217 | 395 | 74.75 | (67.38-82.93) | 75.44 | (68.06-83.62) | #1.48 | (1.31-1.67) | #24.48 | (16.18-32.78) |
| †without | 316475 | 2928 | 37.46 | (36.00-38.97) | 50.96 | (47.92-54.19) | 1 | (1.00-1.00) |  |  |
| **Intraspinal, intraocular, retroperitoneal, intra-articular, pericardial, or intramuscular hemorrhage** | | | | | | | | |  |  |
| Typical |  |  |  |  |  |  |  |  |  |  |
| with | 14986 | 14 | 3.74 | (2.21-6.31) | 3.74 | (2.21-6.31) | #1.97 | (1.14-3.41) | 1.84 | (-0.14-3.81) |
| †without | 322706 | 151 | 1.87 | (1.59-2.20) | 1.9 | (1.59-2.27) | 1 | (1.00-1.00) |  |  |
| Atypical |  |  |  |  |  |  |  |  |  |  |
| with | 21217 | 9 | 1.7 | (0.82-3.49) | 1.7 | (0.82-3.49) | 0.99 | (0.46-2.12) | -0.02 | (-1.33-1.28) |
| †without | 316475 | 156 | 1.97 | (1.68-2.31) | 1.72 | (1.33-2.23) | 1 | (1.00-1.00) |  |  |

*Adjusted by inverse probability of treatment weighting using the propensity score (gender, age, medical utilization, hypertension, myocardial infarction, congestive heart failure, percutaneous coronary intervention, coronary bypass surgery, peripheral vascular disease, cerebrovascular disease, ischemic stroke, transient ischemic attack, hemiplegia or paraplegia, dementia, epilepsy, diabetes mellitus, chronic kidney disease, chronic pulmonary disease, peptic ulcer disease, liver disease, malignancy, anemia, rheumatic disease, human immunodeficiency virus infection, anti-biotics and anti-fungal drugs, anti-epileptics, anti-hypertensives, anti-platelets, bisphosphate, cardiovascular drugs, cyclosporine, glucocorticoid, insulin, lipid lower drugs, nonsteroid anti–inflammatory drugs, proton pump inhibitors, residence, income level, and occupation, see **Tables 1 and 2, and Supplementary Table 3**).

†Without indicates NOAC alone.

#*P* < 0.05, compared with NOAC alone.

**Table S7**. Bleeding risk among AF patients taking apixaban with or without concurrent anti-psychotics.

| **Concurrent medication** | **Person-Quarters with NOAC use** | **No. of Bleeding Events** | **Crude Major Bleeding Incidence Rate (95% CI) per 1000 Person-Years** | | ***Adjusted Incidence Rate (95% CI) per 1000 Person-Years** | | ***Adjusted Rate Ratio (95% CI)** | | ***Adjusted Incidence Rate Difference (95% CI) per 1000 Person-Years** | |
| --- | --- | --- | --- | --- | --- | --- | --- | --- | --- | --- |
| **Major bleeding** | |  |  |  |  |  |  |  |  |  |
| Typical |  |  |  |  |  |  |  |  |  |  |
| with | 4157 | 155 | 148.27 | (126.33-174.03) | 150.51 | (128.61-176.13) | #2.82 | (2.37-3.36) | #97.22 | (73.26-121.18) |
| †without | 85992 | 943 | 44.6 | (41.63-47.78) | 53.29 | (49.27-57.63) | 1 | (1.00-1.00) |  |  |
| Atypical |  |  |  |  |  |  |  |  |  |  |
| with | 6321 | 161 | 102.38 | (86.77-120.81) | 103.75 | (88.12-122.16) | #1.69 | (1.40-2.04) | #42.31 | (24.44-60.18) |
| †without | 83828 | 937 | 45.41 | (42.40-48.63) | 61.45 | (55.75-67.72) | 1 | (1.00-1.00) |  |  |
| **Intracerebral hemorrhage** | |  |  |  |  |  |  |  |  |  |
| Typical |  |  |  |  |  |  |  |  |  |  |
| with | 4157 | 45 | 43.91 | (32.78-58.82) | 43.7 | (32.64-58.52) | #5 | (3.54-7.07) | #34.97 | (22.11-47.83) |
| †without | 85992 | 158 | 7.48 | (6.34-8.82) | 8.73 | (7.26-10.50) | 1 | (1.00-1.00) |  |  |
| Atypical |  |  |  |  |  |  |  |  |  |  |
| with | 6321 | 37 | 23.58 | (17.02-32.66) | 24.05 | (17.41-33.23) | #2.3 | (1.54-3.45) | #13.62 | (5.49-21.75) |
| †without | 83828 | 166 | 8.05 | (6.86-9.44) | 10.44 | (8.11-13.43) | 1 | (1.00-1.00) |  |  |
| **Gastrointestinal hemorrhage** | |  |  |  |  |  |  |  |  |  |
| Typical |  |  |  |  |  |  |  |  |  |  |
| with | 4157 | 107 | 101.24 | (83.27-123.08) | 103.74 | (85.77-125.49) | #2.41 | (1.96-2.96) | #60.66 | (40.63-80.68) |
| †without | 85992 | 756 | 35.74 | (33.08-38.62) | 43.09 | (39.44-47.08) | 1 | (1.00-1.00) |  |  |
| Atypical |  |  |  |  |  |  |  |  |  |  |
| with | 6321 | 123 | 77.97 | (64.24-94.63) | 79.09 | (65.34-95.73) | #1.6 | (1.28-1.98) | #29.51 | (13.59-45.43) |
| †without | 83828 | 740 | 35.83 | (33.16-38.72) | 49.57 | (44.52-55.20) | 1 | (1.00-1.00) |  |  |
| **Intraspinal, intraocular, retroperitoneal, intra-articular, pericardial, or intramuscular hemorrhage** | | | | | | | | | | |
| Typical |  |  |  |  |  |  |  |  |  |  |
| with | 4157 | 3 | 2.89 | (0.93-8.95) | 2.89 | (0.93-8.95) | 2.06 | (0.62-6.81) | 1.49 | (-1.82-4.80) |
| †without | 85992 | 29 | 1.35 | (0.94-1.94) | 1.4 | (0.95-2.07) | 1 | (1.00-1.00) |  |  |
| Atypical |  |  |  |  |  |  |  |  |  |  |
| with | 6321 | 1 | 0.63 | (0.09-4.49) | 0.63 | (0.09-4.49) | 0.43 | (0.06-3.26) | -0.84 | (-2.30-0.62) |
| †without | 83828 | 31 | 1.48 | (1.04-2.10) | 1.47 | (0.87-2.49) | 1 | (1.00-1.00) |  |  |

*Adjusted by inverse probability of treatment weighting using the propensity score (gender, age, medical utilization, hypertension, myocardial infarction, congestive heart failure, percutaneous coronary intervention, coronary bypass surgery, peripheral vascular disease, cerebrovascular disease, ischemic stroke, transient ischemic attack, hemiplegia or paraplegia, dementia, epilepsy, diabetes mellitus, chronic kidney disease, chronic pulmonary disease, peptic ulcer disease, liver disease, malignancy, anemia, rheumatic disease, human immunodeficiency virus infection, anti-biotics and anti-fungal drugs, anti-epileptics, anti-hypertensives, anti-platelets, bisphosphate, cardiovascular drugs, cyclosporine, glucocorticoid, insulin, lipid lower drugs, nonsteroid anti–inflammatory drugs, proton pump inhibitors, residence, income level, and occupation, see **Tables 1 and 2, and Supplementary Table 3**).

†Without indicates NOAC alone.

#*P* < 0.05, compared with NOAC alone.

**Table S8**. Bleeding risk among AF patients taking edoxaban with or without concurrent anti-psychotics.

| **Concurrent medication** | **Person~ Quarters with NOAC use** | **No. of Bleeding Events** | **Crude Major Bleeding Incidence Rate (95% CI) per 1000 Person~Years** | | ***Adjusted Incidence Rate (95% CI) per 1000 Person~Years** | | ***Adjusted Rate Ratio (95% CI)** | | ***Adjusted Incidence Rate Difference (95% CI) per 1000 Person-Years** | |
| --- | --- | --- | --- | --- | --- | --- | --- | --- | --- | --- |
| **Major bleeding** | |  |  |  |  |  |  |  |  |  |
| Typical |  |  |  |  |  |  |  |  |  |  |
| with | 1286 | 36 | 110.88 | (80.12-153.44) | 112.72 | (81.77-155.39) | 1.39 | (0.99-1.94) | 31.4 | (-5.55-68.36) |
| †without | 26353 | 351 | 54.06 | (48.50-60.26) | 81.32 | (71.36-92.66) | 1 | (1.00-1.00) |  |  |
| Atypical |  |  |  |  |  |  |  |  |  |  |
| with | 1619 | 52 | 128.5 | (95.04-173.74) | 131.13 | (97.40-176.54) | #1.55 | (1.09-2.20) | #46.5 | (4.44-88.56) |
| †without | 26020 | 335 | 52.09 | (46.65-58.16) | 84.64 | (70.16-102.10) | 1 | (1.00-1.00) |  |  |
| **Intracerebral hemorrhage** | |  |  |  |  |  |  |  |  |  |
| Typical |  |  |  |  |  |  |  |  |  |  |
| with | 1286 | 6 | 18.66 | (8.40-41.49) | 18.66 | (8.39-41.49) | 2.12 | (0.88-5.10) | 9.85 | (-5.41-25.10) |
| †without | 26353 | 42 | 6.37 | (4.71-8.62) | 8.81 | (6.12-12.70) | 1 | (1.00-1.00) |  |  |
| Atypical |  |  |  |  |  |  |  |  |  |  |
| with | 1619 | 6 | 14.82 | (6.65-33.01) | 14.82 | (6.65-33.01) | 0.98 | (0.33-2.84) | -0.37 | (-16.41-15.66) |
| †without | 26020 | 42 | 6.46 | (4.77-8.73) | 15.19 | (7.48-30.88) | 1 | (1.00-1.00) |  |  |
| **Gastrointestinal hemorrhage** | |  |  |  |  |  |  |  |  |  |
| Typical |  |  |  |  |  |  |  |  |  |  |
| with | 1286 | 29 | 89.59 | (62.51-128.39) | 90.82 | (63.52-129.86) | 1.28 | (0.88-1.86) | 19.92 | (-13.19-53.03) |
| †without | 26353 | 302 | 46.51 | (41.34-52.34) | 70.9 | (61.52-81.71) | 1 | (1.00-1.00) |  |  |
| Atypical |  |  |  |  |  |  |  |  |  |  |
| with | 1619 | 46 | 113.13 | (81.72-156.62) | 115.26 | (83.67-158.77) | #1.7 | (1.18-2.45) | #47.42 | (8.71-86.14) |
| †without | 26020 | 285 | 44.35 | (39.31-50.04) | 67.84 | (57.00-80.74) | 1 | (1.00-1.00) |  |  |
| **Intraspinal, intraocular, retroperitoneal, intra-articular, pericardial, or intramuscular hemorrhage** | | | | | | | | | |  |
| Typical |  |  |  |  |  |  |  |  |  |  |
| with | 1286 | 1 | 3.11 | (0.44-22.05) | 3.11 | (0.44-22.07) | 2.34 | (0.27-19.92) | 1.78 | (-4.42-7.98) |
| †without | 26353 | 7 | 1.06 | (0.51-2.23) | 1.33 | (0.56-3.16) | 1 | (1.00-1.00) |  |  |
| Atypical |  |  |  |  |  |  |  |  |  |  |
| with | 1619 | 0 |  |  |  |  | 0 |  |  |  |
| †without | 26020 | 8 |  |  |  |  | 1 | (1.00-1.00) |  |  |

*Adjusted by inverse probability of treatment weighting using the propensity score (gender, age, medical utilization, hypertension, myocardial infarction, congestive heart failure, percutaneous coronary intervention, coronary bypass surgery, peripheral vascular disease, cerebrovascular disease, ischemic stroke, transient ischemic attack, hemiplegia or paraplegia, dementia, epilepsy, diabetes mellitus, chronic kidney disease, chronic pulmonary disease, peptic ulcer disease, liver disease, malignancy, anemia, rheumatic disease, human immunodeficiency virus infection, anti-biotics and anti-fungal drugs, anti-epileptics, anti-hypertensives, anti-platelets, bisphosphate, cardiovascular drugs, cyclosporine, glucocorticoid, insulin, lipid lower drugs, nonsteroid anti–inflammatory drugs, proton pump inhibitors, residence, income level, and occupation, see **Tables 1 and 2, and Supplementary Table 3**).

†Without indicates NOAC alone.

#*P* < 0.05, compared with NOAC alone.

**Table S9.** Risk of acute appendicitis among patients with AF taking Non–Vitamin K Oral Anticoagulants (NOACs) with or without concurrent anti-psychotics.

| **Concurrent medication** | **Person~ Quarters with NOAC use** | **No. of Events** | **Crude Incidence Rate (95% CI) per 1000 Person~Years** | | ***Adjusted Incidence Rate (95% CI) per 1000 Person~Years** | | ***Adjusted Rate Ratio (95% CI)** | | ***Adjusted Incidence Rate Difference (95% CI) per 1000 Person-Years** | |
| --- | --- | --- | --- | --- | --- | --- | --- | --- | --- | --- |
| **Acute appendicitis** | |  |  |  |  |  |  |  |  |  |
| Typical |  |  |  |  |  |  |  |  |  |  |
| with | 30057 | 6 | 0.8 | (0.36-1.78) | 0.8 | (0.36-1.78) | 0.85 | (0.99-1.94) | -0.15 | (-0.80-0.51) |
| †without | 675464 | 143 | 0.85 | (0.72-1.00) | 0.95 | (0.78-1.15) | 1 | (1.00-1.00) |  |  |
| Atypical |  |  |  |  |  |  |  |  |  |  |
| with | 41658 | 7 | 0.67 | (0.29-1.56) | 0.67 | (0.29-1.56) | 0.89 | (0.37-2.11) | -0.09 | (-0.67-0.50) |
| †without | 663863 | 142 | 0.86 | (0.72-1.01) | 0.95 | (0.78-1.15) | 1 | (1.00-1.00) |  |  |

*Adjusted by inverse probability of treatment weighting using the propensity score (gender, age, medical utilization, hypertension, myocardial infarction, congestive heart failure, percutaneous coronary intervention, coronary bypass surgery, peripheral vascular disease, cerebrovascular disease, ischemic stroke, transient ischemic attack, hemiplegia or paraplegia, dementia, epilepsy, diabetes mellitus, chronic kidney disease, chronic pulmonary disease, peptic ulcer disease, liver disease, malignancy, anemia, rheumatic disease, human immunodeficiency virus infection, antibiotics and antifungal drugs, antiepileptics, antihypertensives, antiplatelets, bisphosphate, cardiovascular drugs, cyclosporine, glucocorticoid, insulin, lipid lower drugs, nonsteroid anti-inflammatory drugs, residence, income level, and occupation; see **Tables 1 and 2, and Supplementary Table 3**).

†without indicates DOAC alone.

**Table S10**. Major bleeding risk among AF patients taking non-vitamin-K antagonist oral anticoagulants (NOACs) with or without concurrent anti-psychotics, after removing bleeding events within the first person-quarter.

| **Concurrent medication** | **Person-Quarters with NOAC use** | **No. of Bleeding Events** | **Crude Major Bleeding Incidence Rate (95% CI) per 1000 Person-Years** | | ***Adjusted Incidence Rate (95% CI) per 1000 Person-Years** | | ***Adjusted Rate Ratio (95% CI)** | | ***Adjusted Incidence Rate Difference (95% CI) per 1000 Person-Years** | |
| --- | --- | --- | --- | --- | --- | --- | --- | --- | --- | --- |
| **Major bleeding** | | | | | | | | | | |
| Typical |  |  |  |  |  |  |  |  |  |  |
| with | 29829 | 936 | 124.27 | (116.23-132.88) | 126.67 | (118.60-135.29) | #2.47 | (2.30-2.65) | #75.39 | (66.98-83.81) |
| †without | 675692 | 7058 | 42.26 | (41.19-43.36) | 51.28 | (49.79-52.80) | 1 | (1.00-1.00) |  |  |
| Atypical |  |  |  |  |  |  |  |  |  |  |
| with | 41478 | 998 | 96.12 | (89.92-102.75) | 97.71 | (91.49-104.35) | #1.66 | (1.54-1.79) | #38.76 | (31.97-45.56) |
| †without | 664043 | 6996 | 42.61 | (41.53-43.73) | 58.94 | (56.62-61.37) | 1 | (1.00-1.00) |  |  |
| **Intracerebral hemorrhage** | | | | | | | | | | |
| Typical |  |  |  |  |  |  |  |  |  |  |
| with | 29829 | 183 | 24.71 | (21.37-28.58) | 24.76 | (21.42-28.62) | #3.58 | (3.05-4.21) | #17.85 | (14.23-21.46) |
| †without | 675692 | 1038 | 6.22 | (5.83-6.62) | 6.91 | (6.44-7.42) | 1 | (1.00-1.00) |  |  |
| Atypical |  |  |  |  |  |  |  |  |  |  |
| with | 41478 | 200 | 19.33 | (16.70-22.36) | 19.71 | (17.05-22.77) | #2.11 | (1.77-2.53) | #10.39 | (7.38-13.40) |
| †without | 664043 | 1021 | 6.22 | (5.84-6.63) | 9.32 | (8.36-10.39) | 1 | (1.00-1.00) |  |  |
| **Gastrointestinal hemorrhage** | | | | | | | | | | |
| Typical |  |  |  |  |  |  |  |  |  |  |
| with | 29829 | 730 | 96.31 | (89.22-103.97) | 98.58 | (91.47-106.25) | #2.31 | (2.13-2.50) | #55.85 | (48.39-63.32) |
| †without | 675692 | 5760 | 34.46 | (33.48-35.46) | 42.73 | (41.35-44.16) | 1 | (1.00-1.00) |  |  |
| Atypical |  |  |  |  |  |  |  |  |  |  |
| with | 41478 | 784 | 75.38 | (69.91-81.28) | 76.57 | (71.09-82.47) | #1.59 | (1.46-1.73) | #28.34 | (22.32-34.37) |
| †without | 664043 | 5706 | 34.71 | (33.73-35.73) | 48.23 | (46.14-50.41) | 1 | (1.00-1.00) |  |  |
| **Intraspinal, intraocular, retroperitoneal, intra-articular, pericardial, or intramuscular hemorrhage** | | | | | | | | | | |
| Typical |  |  |  |  |  |  |  |  |  |  |
| with | 29829 | 23 | 3.08 | (2.05-4.64) | 3.08 | (2.05-4.64) | #1.97 | (1.28-3.02) | #1.52 | (0.24-2.79) |
| †without | 675692 | 260 | 1.54 | (1.36-1.74) | 1.57 | (1.37-1.79) | 1 | (1.00-1.00) |  |  |
| Atypical |  |  |  |  |  |  |  |  |  |  |
| with | 41478 | 14 | 1.35 | (0.77-2.36) | 1.35 | (0.77-2.36) | 0.98 | (0.54-1.76) | #-0.03 | (-0.84-0.77) |
| †without | 664043 | 269 | 1.62 | (1.43-1.83) | 1.38 | (1.14-1.68) | 1 | (1.00-1.00) |  |  |

*Adjusted by inverse probability of treatment weighting using the propensity score (gender, age, medical utilization, hypertension, myocardial infarction, congestive heart failure, percutaneous coronary intervention, coronary bypass surgery, peripheral vascular disease, cerebrovascular disease, ischemic stroke, transient ischemic attack, hemiplegia or paraplegia, dementia, epilepsy, diabetes mellitus, chronic kidney disease, chronic pulmonary disease, peptic ulcer disease, liver disease, malignancy, anemia, rheumatic disease, human immunodeficiency virus infection, anti-biotics and anti-fungal drugs, anti-epileptics, anti-hypertensives, anti-platelets, bisphosphate, cardiovascular drugs, cyclosporine, glucocorticoid, insulin, lipid lower drugs, nonsteroid anti–inflammatory drugs, proton pump inhibitors, residence, income level, and occupation, see **Tables 1 and 2, and Supplementary Table 3**).

†Without indicates NOAC alone.

#*P* < 0.05, compared with NOAC alone.
